# The zebrafish gut microbiome influences benzo[a]pyrene developmental neurobehavioral toxicity

**DOI:** 10.1038/s41598-024-65610-3

**Published:** 2024-06-25

**Authors:** Keaton Stagaman, Alexandra Alexiev, Michael J. Sieler, Austin Hammer, Kristin D. Kasschau, Lisa Truong, Robyn L. Tanguay, Thomas J. Sharpton

**Affiliations:** 1 Department of Microbiology, Oregon State University, 226 Nash Hall, Corvallis, OR 97331 USA; 2 Sinnhuber Aquatic Research Laboratory, Department of Environmental and Molecular Toxicology, Oregon State University, Corvallis, OR USA; 3 Department of Statistics, Oregon State University, Corvallis, OR USA

**Keywords:** Zebrafish, Benzo[a]pyrene, Development, Behavior, Neurophysiology, Toxicity, Gut microbiome, Microbiology, Microbial communities

## Abstract

Early-life exposure to environmental toxicants like Benzo[a]pyrene (BaP) is associated with several health consequences in vertebrates (i.e., impaired or altered neurophysiological and behavioral development). Although toxicant impacts were initially studied relative to host physiology, recent studies suggest that the gut microbiome is a possible target and/or mediator of behavioral responses to chemical exposure in organisms, via the gut-brain axis. However, the connection between BaP exposure, gut microbiota, and developmental neurotoxicity remains understudied. Using a zebrafish model, we determined whether the gut microbiome influences BaP impacts on behavior development. Embryonic zebrafish were treated with increasing concentrations of BaP and allowed to grow to the larval life stage, during which they underwent behavioral testing and intestinal dissection for gut microbiome profiling via high-throughput sequencing. We found that exposure affected larval zebrafish microbiome diversity and composition in a manner tied to behavioral development: increasing concentrations of BaP were associated with increased taxonomic diversity, exposure was associated with unweighted UniFrac distance, and microbiome diversity and exposure predicted larval behavior. Further, a gnotobiotic zebrafish experiment clarified whether microbiome presence was associated with BaP exposure response and behavioral changes. We found that gut microbiome state altered the relationship between BaP exposure concentration and behavioral response. These results support the idea that the zebrafish gut microbiome is a determinant of the developmental neurotoxicity that results from chemical exposure.

## Introduction

Our understanding of the factors that drive behavioral development remains elusive. While genetics certainly play a role^1,2^, increasing attention has been placed on how exposure to environmental chemicals determines early life behavioral outcomes, especially given the recent and rapid rise in neurodegenerative disorders^3–5^. In particular, researchers have uncovered a variety of environmental chemicals that impair neurophysiological and behavioral development^3,4,6–12^. However, our understanding of how these chemicals elicit such effects is incomplete. Recent work has hypothesized that the gut microbiome may act as a critical determinant of how environmental chemicals affect behavioral development^13–23^. For example, the diverse metabolic capabilities of gut microbiota may interact with chemicals to produce diverse metabolic conjugates that directly interact with host tissues, possibly mediating the toxicity of the chemical^14,22–27^. Additionally, environmental chemicals can affect the abundance and metabolic capacity of taxa in the gut^15,28^. This in turn alters the microbiome’s assembly and functional contribution to neurodevelopment via the gut-brain axis^29–37^, indicating that exposure may elicit neurotoxicity through dysbiosis. Prior observations offer a strong rationale for the overarching hypothesis that the gut microbiome is a determinant of neurotoxicity through these or alternative mechanisms in certain model compounds.

One such compound of emergent interest is benzo[a]pyrene (BaP), which remains under-investigated in regard to the link between developmental neurotoxicity and the gut microbiome. BaP, like most other polycyclic aromatic hydrocarbons (PAH), is a widely distributed environmental pollutant^38,39^. It is produced by various natural and anthropogenic processes, primarily through incomplete combustion of organic material (e.g., coal burning, wildfires)^38,39^. Humans are also frequently exposed to BaP through their diet; the toxicant is found at higher levels in oft-consumed foods like cereals and fried, roasted, or smoked meats compared to other foods ^39,40^. BaP itself is the precursor to a variety of toxic and carcinogenic compounds and thus requires biotransformation to elicit toxic effects downstream^39^. Although many of these mechanisms are well-characterized, sources of variation in physiological response to BaP remain unclear. Earlier work has shown that members of the microbiota can metabolize BaP, and the gut microbiome itself exhibits high inter-individual variation in both taxa and metabolic capacity when exposed to BAP^16,34,41^. Thus, evidence from existing literature suggests that gut microbes may interact with environmental chemicals in ways that cause varied downstream toxic effects.

Of particular concern is the developmental effects of BaP, which persists in tissues including breast milk and placenta, raising concern that humans are subject to frequent early life exposure that affects their lifelong health^42^. Further, BaP receives new-found attention as a chemical that impairs behavioral development—epidemiological studies connect parental, prenatal, and early postnatal exposure to childhood behavioral impairment and hyperactivity^43^. In animal models, notably zebrafish (*Danio rerio*), developmental exposure to this endocrine disruptor affects serotonin signaling and oxidative stress in the brain to yield hyperactivity and anxiety in juveniles, along with other detrimental physiological effects^3,44–50^. Specifically, zebrafish exposed to increasing concentrations of BaP during development exhibited hyperactivity in response to light stimulus at various life stages (embryonic, larval, and adult)^48^. This relationship holds true until the chemical begins to elicit additional toxic effects that reduce zebrafish motility, which occurs between 5 and 10 µM BaP. Despite the evidence implicating BaP in behavioral impairment and abnormal development, we do not know how it does so.

We hypothesize that one route through which BaP impairs behavior development is via the gut microbiome. Prior observations about the link between BaP and microorganisms support this hypothesis. For example, humans exposed to BaP exhibit altered gut metatranscriptomes^51^ and adult mice orally exposed to BaP experience changes in the composition of their gut microbiota^52^, which collectively suggests that the microbiome is sensitive to BaP exposure. These observations are also consistent with in vitro assays, which show that gut microbial taxa are differentially sensitive to BaP exposure^51^. Further, the effects of BaP on gut microbiota^53,54^ could result in alterations to how the gut microbiome interacts with the gut-brain axis to drive neurodevelopment. Additionally, some gut microbes have the metabolic capacity to metabolize BaP into diverse conjugates, which may elicit differentially toxic effects^14,55^. BaP’s link to behavior development is also highly variable in animal models^56^. This variation could result from the fact that metabolism of BaP can be highly variable among individuals^57^ combined with the fact that the gut microbiome is highly individual in humans and other vertebrates^58,59^. It follows that variation in the composition of the gut microbiome could underlie the variation in the effects of BaP exposure.

**Figure 1:**
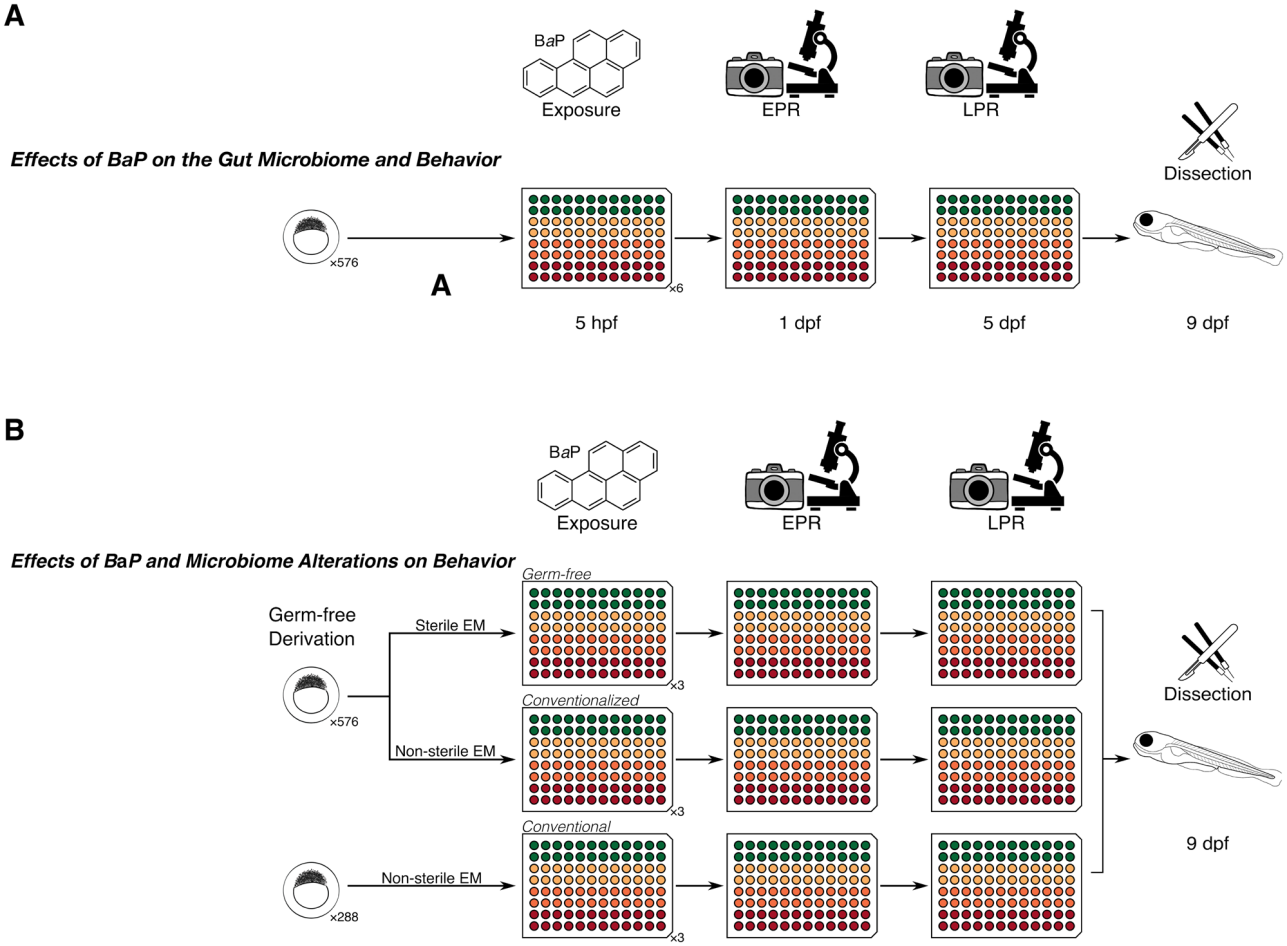
Experimental design for the study. (**A**) shows the design to test whether there was an effect of BaP and the gut microbiome on behavior in zebrafish. Embryos were exposed to three different concentrations (noted by the colors) in 96-well plates as shown, then measured via EPR, allowed to grow to the larval stage, and LPR behavior assays. Larvae were subsequently dissected for gut microbiome data. (**B**) shows the study design for the effect of gut microbiome presence on the relationship between BaP and behavior. Similarly, colors represent a gradient of BaP exposures that were applied to gnotobiotic, CV, and CVZ zebrafish embryos in 96-well plates and EPR, LPR, and microbiome data collected. This study design allows us to determine not only the associations between BaP, gut microbiome, and behavior, but also the combined effect of BaP and gut microbiome state on behavior.

Our goal was to determine if embryonic exposure to BaP affects the assembly of the gut microbiome, and whether the gut microbiome influences the neurotoxicity that results from this chemical exposure (Fig. 1). Given that BaP exposure in embryonic zebrafish impairs behavior, we hypothesized that this exposure disrupts the normal assembly of the zebrafish gut microbiome to result in a community that drives maladaptive larval behavior. We reasoned that if this hypothesis is correct, then we should observe that exposure to BaP impacts the diversity or composition of the larval gut microbiome (Fig. 1A). Additionally, the community that assembles in the gut should associate with larval fish behavior (Fig. 1A). Moreover, we would expect that exposing germ-free fish to BaP should result in different behavior responses to exposure as compared to fish that carry microbiomes (Fig. 1B). Therefore, we exposed embryonic zebrafish to environmentally relevant concentrations of BaP (0, 0.2523, 1.2615, and 2.523 µg/mL) and measured their behavioral response (distance moved) to light stimulus at the embryonic and larval stages. After behavioral measurements, we sampled their intestinal microbiomes through gut dissection, DNA extraction, and sequencing, to assess the relationships between BaP exposure, behavioral outcomes, and microbiome diversity and composition. In addition to using environmentally relevant BaP concentrations for exposure, we utilized a non-inbred zebrafish line, that is, a non-isogenic population to represent an environmentally relevant level of genetic diversity more closely. Overall, our results support the overarching hypothesis that the gut microbiome is a determinant of the impact of BaP on larval fish behavior.

## Results

### BaP disrupts zebrafish behavior development

**Figure 2:**
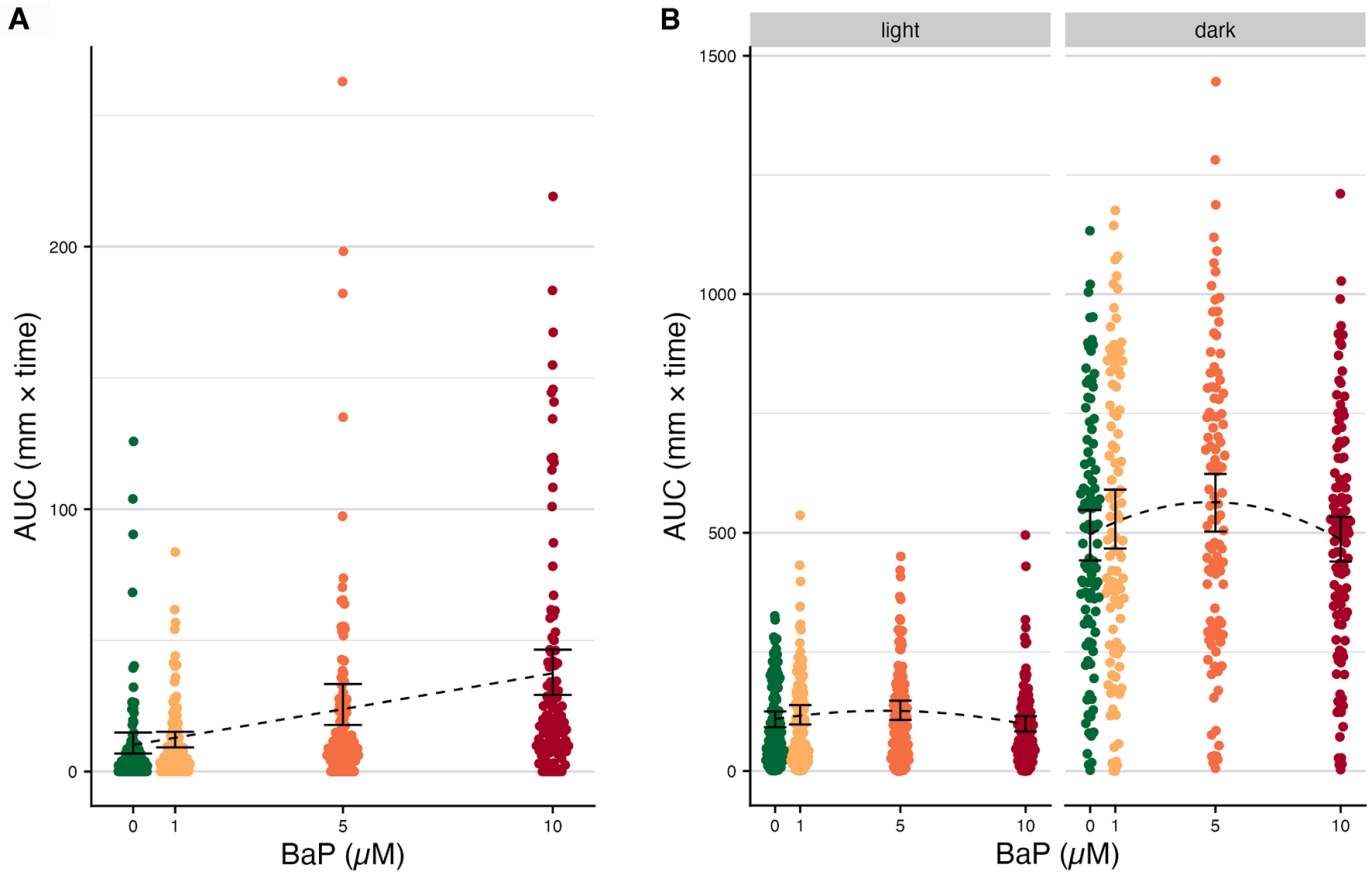
Behavior assay results (**A**) Embryonic photomotor response (EPR) movement data (n = 573). Areas under the curve (AUCs) for the movement curves from panel A, measured only in the window between the dotted lines. Black error bars indicate the 95% C.I.s for the mean AUC per BaP exposure level. The black dotted line indicates the estimated association from linear regression. (**B**) Larval photomotor response (LPR) movement data (n = 573). AUCs for the movement curves from panel A, split by cycle (light versus dark). Black error bars indicate the 95% C.I.s for the mean AUC per BaP exposure level. The black dotted line indicates the estimated association from polynomial linear regression.

Prior work demonstrated that exposure to BaP alters embryonic and larval behavior^48^, so we first sought to confirm that we observe similar effects in our study. Following this prior work, we assessed response to a light pulse trigger in embryos using the embryonic photomotor response (EPR) assay^48^. Consistent with Knecht et al., 2017, we found that exposure to BaP induced hyperactivity following a pulse of light; mean embryonic movement increased significantly with BaP exposure for embryos exposed to 5 and 10 µM BaP relative to no exposure (*p* = 0.00266 and *p* <  < 0.001, respectively) (Fig 2A, Supp. Fig 1A; Supp. Table 1).

Additionally, we assessed the effect of embryonic BaP exposure in five-day-old larval zebrafish using the larval photomotor response (LPR) assay^48,56^. Briefly, this assay subjects larvae (n = 573) to four epochs (24 min) of light and dark cycles and measures the larvae’s movement during each part of the cycle. Light and dark cycles represent two distinct behaviors in zebrafish; the typical response from zebrafish is to exhibit low total movement in the light and increased movement in the dark following the transition of light to dark^60–62^. Atypical behavior is generally when light cycle movement is higher (hyperactive) and/or dark cycle movement is higher or lower (hypoactive) than normal, which has been recorded previously in other zebrafish exposure studies^60–63^. For our analyses, we quantified these measures of behavior for each individual by summing the area under the curve (AUC) of total movement for each cycle. Again, consistent with prior work^48,56^, we found a significant effect of BaP exposure on LPR AUC values (Supp. Table 2) for both the light (*p* = 0.044) and dark (*p* = 0.029) cycles, but this relationship was not linear (Fig. 2B, Supp. Figure 1B). We repeated this analysis using another approach often used for summarizing behavior from these data—the differential entropy statistic^64,65^—to find that our results are robust to the metric utilized and remain consistent with prior work ^48,56^ (Supp. Fig. 2). Going forward, we used the AUC metric to represent behavior because it affords measures of behavior at the individual level, unlike differential entropy which aggregates behavior measures across cohorts. These individual measures are important for analytical integration with individual microbiome measures in subsequent analyses.

### BaP affects larval zebrafish microbiome diversity and composition

**Figure 3:**
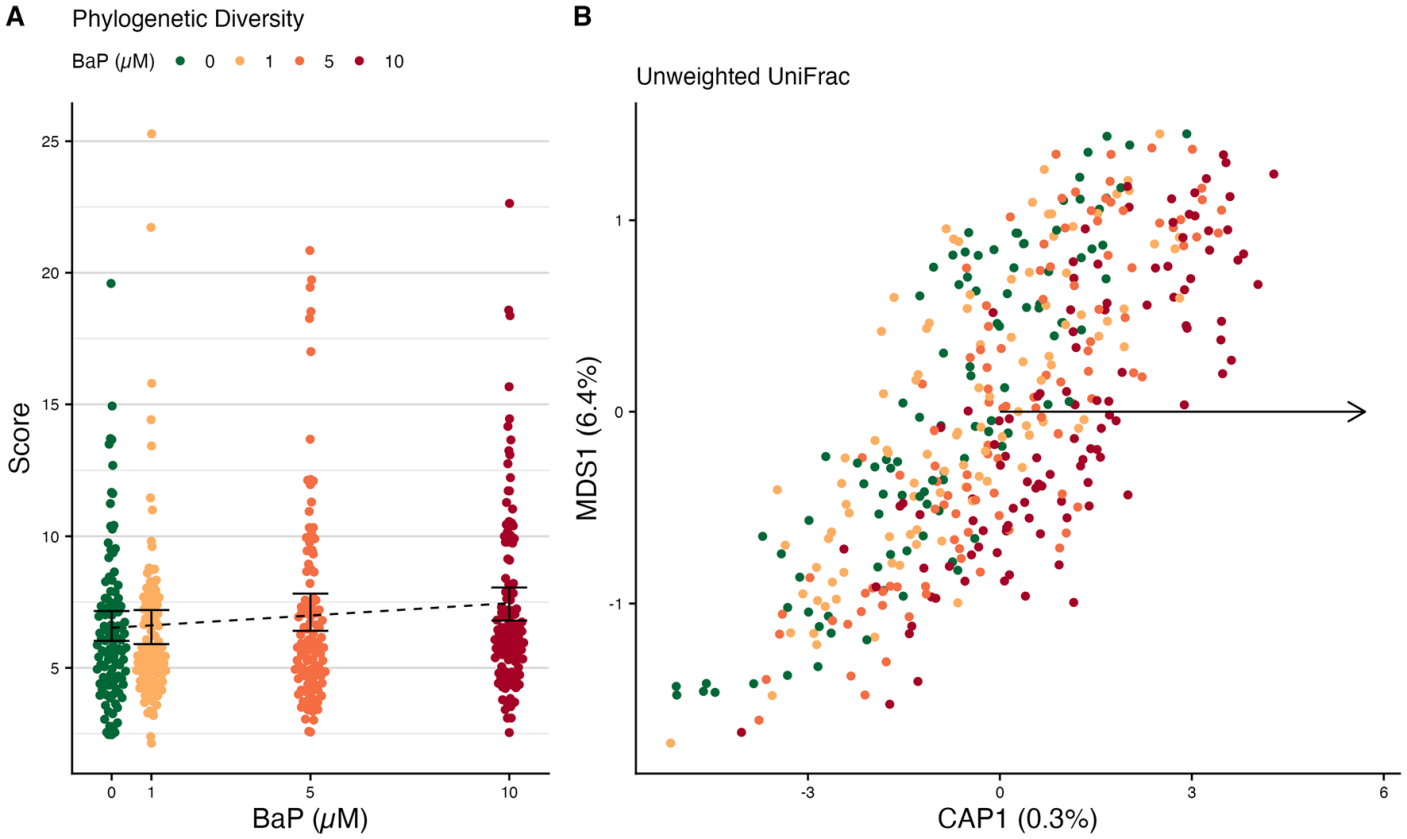
Differences in larval microbiome diversity and composition by BaP exposure. (**A**) Phylogenetic diversity of larval microbiomes. Black error bars represent bootstrapped 95% C.I.s around the means; the dotted black line represents the estimated trend line from regression. (**B**) Unweighted UniFrac beta-diversity dbRDA ordination of larval microbiomes. Points are colored by BaP exposure. The black arrow indicates the direction of the trend in BaP exposure in ordination space. Percentages in the axis labels indicate the percent variance in microbiome composition explained by each axis.

We tested whether BaP exposure has an effect on the diversity and composition of the larval zebrafish microbiome. Here, we evaluated 9 dpf larvae because this represents the earliest developmental timepoint for which we can reliably dissect intestinal tissue from the host. We found associations between BaP exposure and three of the five alpha-diversity metrics we assessed: Chao1, phylogenetic diversity (Fig. 3A), and ASV richness (Supp. Table 3). The other two alpha-diversity metrics, Shannon and Simpson, were not significant. Collectively, these results suggest that as concentrations of BaP exposure increase, as do the number of taxa present in the larval zebrafish intestine, but the rank abundance distribution of microbial taxa in the community is not affected. The additional taxa in the intestine of larval zebrafish exposed to higher doses of BaP are not closely related to other taxa present in the unexposed microbiomes (Fig. 3B and Supp. Fig. 3), possibly indicating that there are taxa that metabolically utilize BaP (or its derivatives) in the zebrafish gut.

We calculated four beta-diversity metrics to assess whether BaP affects microbiome composition: Canberra, Sørensen, weighted UniFrac, and unweighted UniFrac (Fig. 3B). Of these, BaP exposure was associated with the unweighted UniFrac distance metric at a false discovery rate of 0.09 (Supp. Table 4). This metric considers the presence and absence of taxa, as well as their phylogenetic relationships, so it is particularly sensitive to the addition or loss of rare and phylogenetically distinct taxa. These beta-diversity results are concordant with the alpha-diversity results and together suggest that BaP exposure primarily drives an increase in low-abundance, distantly related taxa in the larval zebrafish intestinal microbiome.

To clarify the set of taxa that potentially underlie the association between BaP exposure and the gut microbiome, we utilized a random forest model to identify taxa that best explain the variation in BaP exposure across individuals. In particular, we established a classification model to determine how well we could predict BaP exposure from taxon abundance and to reveal the particular taxa that putatively link to BaP exposure concentration. We assessed how well two different taxa sets could predict exposures: an ASV-only set and an aggregated set in which we not only included abundances of individual ASVs, but also their higher-level taxonomic assignments (e.g., genus, family, class, etc.). Doing so had the effect of reducing the dimensionality of the total number of ASVs or phylotypes we subsequently linked to BaP concentration through statistical regression. We trained random forest models using 70% of the data and evaluated the predictive power of the models using the remaining 30% of the data. The results of our classification model were modest, but better than those expected by chance: the receiver operating characteristic (ROC) AUC was 0.54, meaning it accurately classified samples ~ 54% of the time (regardless of the taxa set), which is better than the 25% expected by random chance for four possible classes (the confusion matrices can be found in Supp. Table 5). We identified the taxa that were best at predicting BaP exposure for the aggregated model and plotted the abundance by exposure level for the top 20 of these taxa (Supp. Fig. 4). Overall, our results indicate that BaP elicits modest effects on the larval zebrafish gut microbiome. This result could be attributed to the myriad metabolic products that can be produced from BaP or the genetic diversity of the zebrafish population we sampled. Additionally, microbiome assembly is inherently subject to stochastic forces that can drive priority effects, which in turn can yield extensive variation among individuals^66,67^. Regardless, our finding suggests that larval zebrafish microbiome assembly is subject to extensive variation, but that developmental BaP exposure is a factor that influences the structure of the assembling zebrafish microbiome.

### Microbiome diversity and BaP exposure predict larval behavior

**Figure 4:**
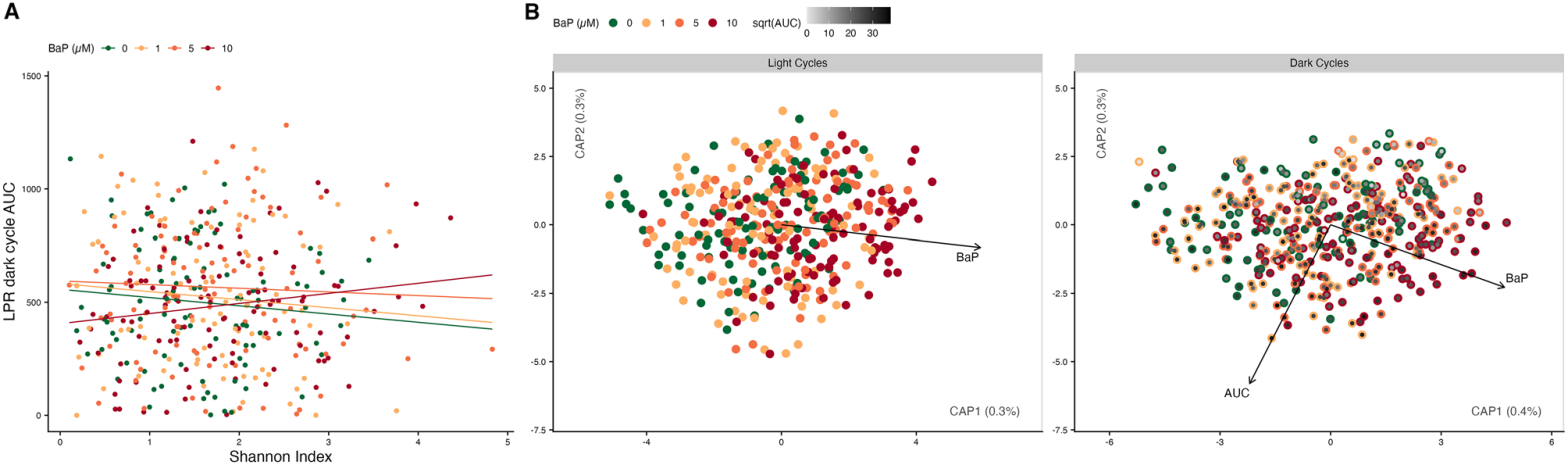
BaP effect on microbiome diversity metrics and behavior. (**A**) Scatter plot of alpha-diversity scores (Shannon index) on the x-axis and LPR dark cycle AUCs (larval activity) on the y-axis. Lines indicate relationships estimated from mixed-effects linear regression models, the interaction between Shannon scores and BaP exposure is significant (*p* = 0.038). Points and lines are colored by BaP exposure. (**B**) Distance-based redundancy analysis (dbRDA) ordination of unweighted UniFrac scores comparing microbiome composition between samples. Each point represents a sample, the closer the samples, the more alike their microbiome composition is according to the distance metric. Black arrows indicate the direction of greatest change in the indicated covariates. Only statistically significant relationships are shown. In the left panel, ‘light cycles’, points are colored by BaP exposure. In the right panel, ‘dark cycles’, the outer edges of the points are colored by BaP exposure while the center of the points are colored by the square-root (for visualization purposes) of the LPR AUC for each sample.

**Figure 5:**
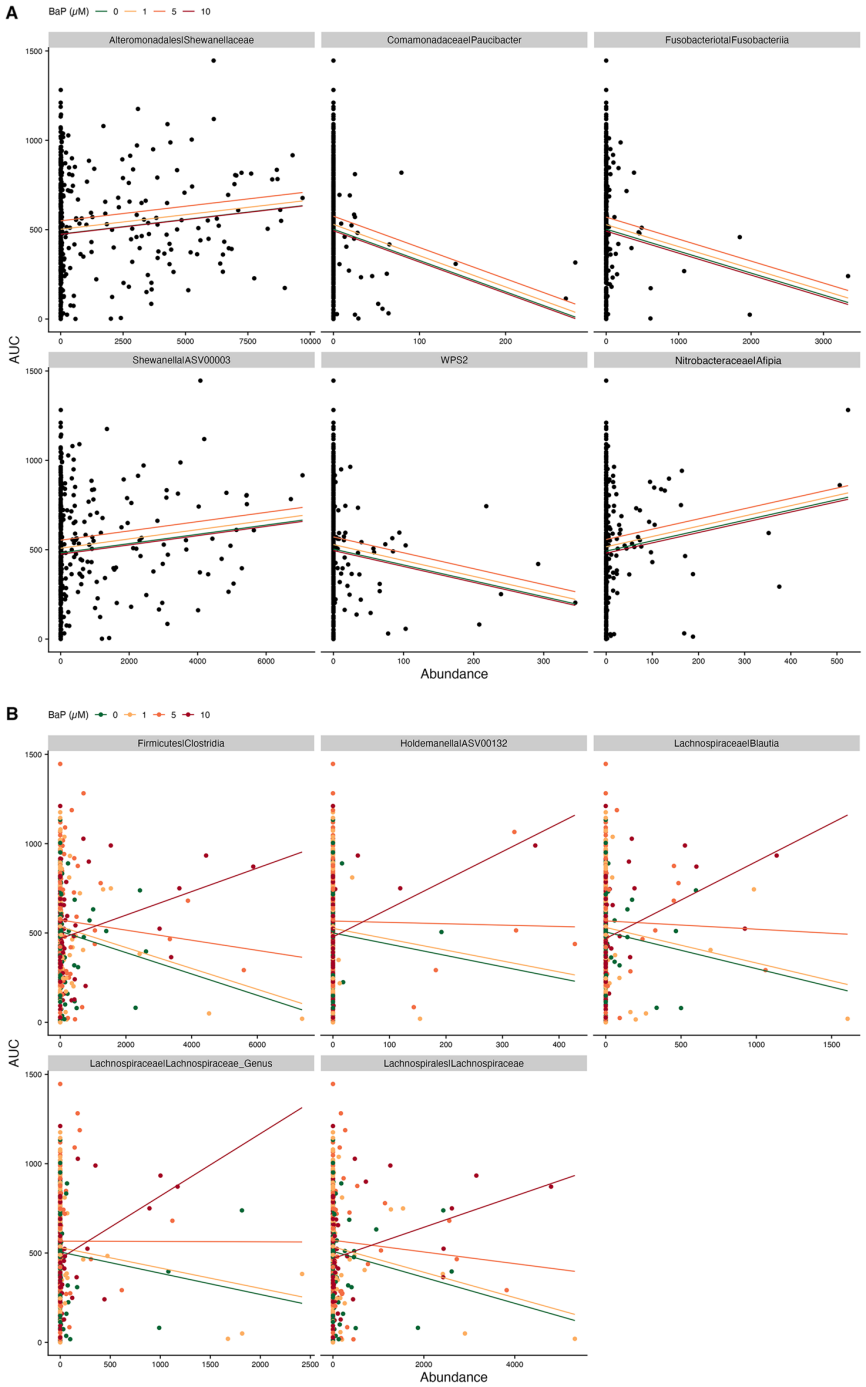
Scatter plots of taxon abundance by LPR dark cycle AUCs. (**A**) Examples of taxa with significant main effects, i.e., the predicted relationship is the same for all BaP exposures. (**B**) Examples of taxa with significant interactions, i.e., the predicted relationship is different for different levels of BaP exposure. For both panels, significance indicates *p* < 0.05., and lines indicate estimated regression relationships from models, colored by BaP exposure.

Given that BaP affects zebrafish behavior and their intestinal microbiome, and that the microbiome affects their behavior, we then utilized microbiome and BaP exposure data in conjunction (as opposed to independently) to predict zebrafish larval behavior. More specifically, we tested whether there were interactions between BaP and microbiome diversity in predicting LPR AUCs. Only one metric, the Shannon index, interacted with BaP exposure in predicting LPR AUCs (Fig. 4A; Supp. Table 6). As Fig. 5A shows, the relationship between alpha-diversity within specific genera and larval activity is negative for most BaP exposure levels. However, at 10 µM, this relationship flips and becomes positive. That is, greater microbiome diversity changes from associating with lower larval activity to associating with greater larval activity at the highest concentration we exposed fish to.

We predicted beta-diversity from LPR AUC values and BaP exposure using PERMANOVA models. We built full models, which included an interaction term between the predictors, then optimized the models using the Akaike information criterion (AIC). For two of the four beta-diversity metrics we assessed, only LPR AUC was retained as a predictor in the optimal model. The results were modest and did not show any significant patterns between beta diversity and BaP interacting with behavior after *p*-value correction. However, the main effects of BaP and behavior were closer to significance compared to the interaction term in the optimal model for unweighted UniFrac (Fig. 4B; Supp. Table 7). Beta diversity in the light cycle was associated with only BaP dosage, whereas in the dark was associated with BaP dosage and AUC. As with alpha-diversity, this implies there are orthogonal associations between larval zebrafish microbiome composition and both behavior and BaP exposure.

To determine which taxa putatively underlie the variation in microbiome composition as a function of BaP and larval zebrafish behavior, we again used random forest to prioritize candidate taxa that were subsequently statistically evaluated using multivariate regression. In this case, we used random forest regression models to predict LPR AUC values from taxon abundances. We created different models for light vs dark cycles, and we again utilized both ASV-only and aggregate abundance data sets as predictors. Both taxon sets performed similarly: the light cycle random forest models an error rate of 16% and the dark cycle had an error rate of 20%, relative to the max AUC for each cycle (Supp. Table 8 shows expanded regression model metrics).

Utilizing the significantly important taxa (n = 503) from the random forest models, we wanted to determine if there were specific taxa that could significantly predict zebrafish behavior in either BaP-dependent or -independent manner. To do so, we generated multivariate linear regression models, predicting LPR AUC values from individual taxon abundances. We identified a number of taxa that were associated with increased LPR activity, regardless of BaP exposure, and could be investigated further in the future. For the light cycle, these included an ASV (ASV00154) in the *Methylobacterium–Methylorubrum* genus, an ASV (ASV00071) in the *Methylophyilus* genus, and an ASV (ASV00006) in the *Pseudomonas* genus (Supp. Fig. 5, Supp. Table 9). The first two of these, while statistically significant, may primarily be driven by a few outliers. *Pseudomonas*, however, appears to be a robust association worthy of further investigation in subsequent studies.

For the dark cycle, we identified an ASV (ASV00003) in the *Shewanella* genus and the Shewanellaceae family, as well as the *Afipia* (Nitrobacteraceae) genus as having positive associations with greater larval activity. Notable taxa assigned to the Shewanellaceae family, including the genus *Shewanella* (ASV00003) had positive associations with LPR dark cycle movement, regardless of BaP exposure level. These taxa are intriguing as environmental samples of *Shewanella* species have been shown to metabolize PAHs, including BaP^68,69^. Furthermore, a study of fecal microbiome transplants from humans with IBS (and healthy controls) to mice found that microbial taxa assigned to the Shewanallaceae family were associated with anxiety-like behavior in the mice^70^. We also identified a number of taxa associated with reduced LPR AUCs, in particular the genera *Paucibacter* (Comamonadaceae), *Undibacterium* (Oxalobacteraceae), *Novosphingobium* (Sphingomonadaceae); the classes Bdellovibrionia (Bdellovibrionota) and Fusobacteriia (Fusobacteriota); and the proposed phylum WPS2 (Fig. 5A; Supp. Fig. 5; Supp. Table 9). *Paucibacter* (Comamonadaceae) has been strongly linked to the core microbiome of healthy zebrafish across multiple exposure studies^17,71^.

We also identified taxa whose association with larval activity depended on BaP exposure (i.e., a significant interaction term). For the light cycle, we identified just one taxon, ASV00067, in the genus *Pseudoxanthomonas*, which had a positive relationship with LPR AUC value for all BaP exposure levels, except 10 µM (Supp. Fig. 6; Supp. Table 9). ASV00067 comes from a group that is known to biodegrade some pollutants in plants^72^. This ASV had a significant interaction with BaP exposure wherein its abundance was positively associated with light cycle movement for concentrations below 10 µM, and negatively associated with light cycle movement for the 10 µM treatment, although, this relationship does appear to be driven by outliers (Supp. Fig. 6). For the dark cycle, we identified a number of related taxa that had a negative association with larval activity at 0 µM BaP, but this relationship became increasingly more positive with increasing BaP exposure (Fig. 5B; Supp. Fig. 6; Supp. Table 9). These taxa include two genera, *Blautia* and an undefined genus, both in the Lachnospiraceae family: the Lachnospiraceae family (Clostridia), and the Clostridia class (Firmicutes). These taxa, perhaps, represent microbiota that directly contribute to the effects of BaP on zebrafish behavior either through the metabolism of BaP that produces other toxic derivatives, or through the production of other metabolites that exacerbate the direct effects of BaP. Members of the Lachnospiraceae family have been linked to numerous behavior outcomes in both mice and humans, though these relationships can be quite complex and difficult to predict^73^. Furthermore, in a study of bioremediation of rice paddies polluted with BaP, Lachnospiraceae bacteria were in the top five associated with BaP removal^74^. These taxa are of interest because their relationship with zebrafish behavior do not seem to be straightforward. Although there are many caveats to using abundance-based methods to identify taxa associated with variables of interest, it is also a notable method to drive discovery and new hypotheses. In vitro and in vivo experiments can be informed by this sort of analysis and are the next step in confirming whether a connection does truly exist between ASVs and BaP neurotoxicity. Confirming whether Lachnospiraceae and other taxa can affect behavior and by what mechanisms remains an important area of study and would be applicable not just to zebrafish, but vertebrate and environmental health as a whole.

### Broad-scale changes to the microbiome affect the relationship between BaP and behavior

**Figure 6:**
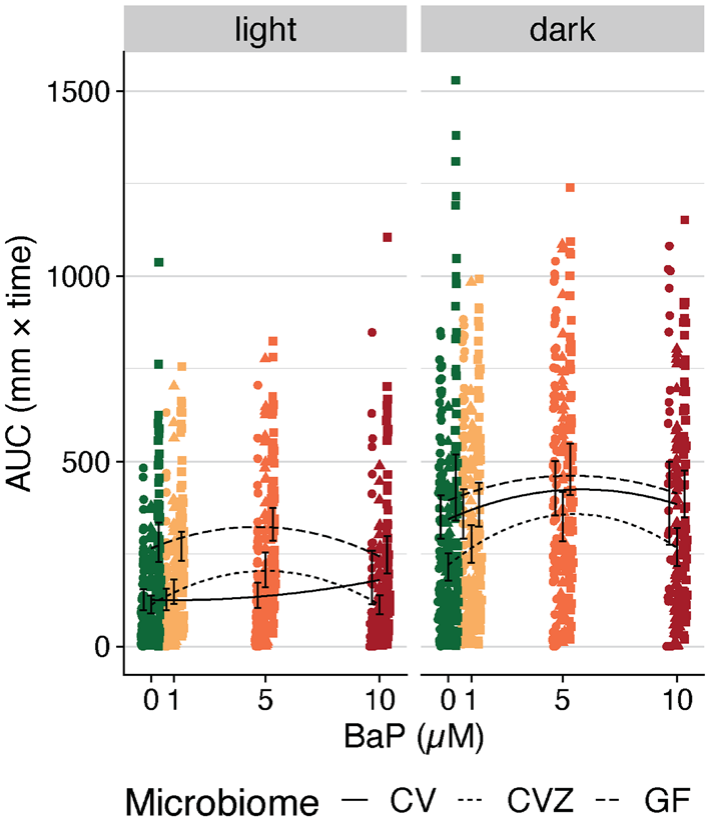
LPR movement areas under the curve (AUCs) split by light and dark. Colors represent each BaP exposure level (in µM). The black lines indicate the estimated association from polynomial linear regression, where line type corresponds to the microbiome’s state in a fish (i.e., conventional (CV), conventionalized (CVZ), or germ free (GF)). Black error bars indicate the 95% C.I.s for the mean AUC per BaP exposure level and microbiome treatment. For the light cycle, GF sample AUCs are significantly higher than CV AUCs, and there is no significant difference between CVZ AUCs and CV AUCs. There is a significant interaction between microbiome state and the second order of BaP concentration (Supp. Table 8). For the dark cycle, CVZ sample AUCs are significantly lower than CV AUCs, and there is no significant difference between GF AUCs and CV AUCs. There is no significant interaction term between microbiome state and BaP in a polynomial linear regression (Supp. Table 8).

To determine what role, if any, the microbiome plays in influencing the relationship between BaP exposure and larval fish behavior, we used germ-free fish to test the effect of modulating the presence of the microbiome of the behavior of fish exposed to BaP. In particular, we measured how behavioral response varies as a function of embryonic BaP across three groups of fish that differ in their microbiome state: (1) those that were conventionally raised (CV) and thus carried a typical microbiome, (2) those that were germ-free (GF) and thus carry no microbiome, and (3) those that were derived germ-free but subsequently conventionalized (CVZ) through exposure to non-sterile zebrafish growth media^75^. This final group is an important experimental component in our study because CVZ fish carry an embryo-media-associated microbiome and thus enable accounting for the effect of the GF derivation process on outcomes (Fig. 6A). We also conducted these comparisons across the same range of BaP exposure concentrations described previously (0, 1, 5, 10 uM). We then used polynomial regression to determine if behavior varies as a function of microbiome state, either BaP concentration or the square of this concentration, or the interaction between microbiome state and these concentration terms.

Our analysis found that microbiome state significantly impacts zebrafish behavior as well as the effect of BaP exposure on behavior, with slight nuances between the light versus dark cycles. We began by considering the light cycle behavioral data. In unexposed zebrafish, GF larvae exhibit significantly higher activity relative to CV larvae across light cycles (Fig. 6B, “light” panel; Supp. Table 3). Looking next across increasing BaP concentration, the slope of the curve in CV larvae is higher compared to GF and CVZ larvae (Fig. 6B, “light” panel). Taken together, this is consistent with the results of the regression model, in which the interaction between microbiome state and BaP concentration were significant (Supp. Table 3). The model indicates that microbiome state does alter the slope of the relationship between behavior and BaP exposure. BaP itself is also not a particularly strong neurotoxicant, so it is notable that we find a signal of gut microbiome and behavior response despite this. Furthermore, this observation implies that the state of the microbiome impacts how BaP exposure relates to fish behavior during light cycles of the LPR test, and therefore demonstrates that the microbiome defines the impact of BaP neurotoxicity. We next evaluated the dark cycle behavioral data. In unexposed zebrafish, GF and CV larvae exhibit significantly higher activity relative to CVZ larvae across dark cycles (Fig. 6B, “dark” panel; Supp. Table 10). While BaP concentration, as well as the square of this concentration, associates with activity as expected, this association does not appear to be affect by the microbiome state of the fish (Supp. Table 10). In other words, microbiome state and BaP exposure both affect dark cycle zebrafish behavior, but microbiome state does not alter the f the relationship between behavior and BaP exposure (or vice versa). It is not yet evident which specific taxa elicit this effect or how members of the microbiota might interact with BaP to affect behavior, and this should be investigated further in future experiments. However, our findings do support that the whole community in the gut determines neurotoxicity from BaP.

CVZ larvae additionally exhibited hypoactivity relative to CV larvae during the dark cycles. GF and CVZ larvae underwent the germ-free derivation process, but CVZ larvae were subsequently colonized with the microbiota in EM. If the germ-free derivation process affected larval behavior significantly, we would expect both GF and CVZ larvae trends to be in the same direction. Thus, differences in response between these two experimental groups relative to CV larvae is not simply due to the effects of the germ-free derivation process.

## Discussion

Emerging research indicates that the manner in which the microbiome assembles in the gut can affect the early-life development vertebrates, including their neurodevelopment^30,31,66,67,76–78^. We hypothesized that exposure to the environmental pollutant BaP could impact gut microbiome assembly to influence behavior development in zebrafish. To determine the validity of this hypothesis, we evaluated how embryonic exposure to BaP impacts the assembly of the zebrafish gut microbiome and whether the gut microbiome associates with the effect of BaP on embryonic and larval zebrafish photomotor function. Overall, the present study supports this hypothesis by finding that (1) BaP exposure perturbs gut microbiome assembly in a dose-dependent fashion, (2) the composition of the microbiome that assembles in the gut explains larval zebrafish behavior, and (3) the gut microbiome impacts how embryonic BaP exposure affects behavioral development in zebrafish. These observations indicate that environmental chemical exposure may influence vertebrate neurodevelopment by impacting gut microbiome assembly and hold important implications for our understanding of the underlying mechanisms of neurodevelopmental disorders.

Prior work observed that the gut microbiomes of mice are sensitive to BaP, as are specific gut isolates: BaP is toxic to some gut microbiota, whereas others are able to resist or even degrade the chemical^52^. However, to date, no study has evaluated how early-life BaP exposure impacts the assembly of the gut microbiome through early development in zebrafish. We found that embryonic BaP exposure perturbs microbiome assembly (in terms of alpha- and beta-diversity) in the zebrafish gut in a dose-dependent manner. In both cases, this dose-dependent effect was only observed using unweighted metrics (i.e., metrics that do not consider the abundance of taxa), indicating that the effect of BaP on the microbiome principally manifests among the rarer taxa in the community. Past studies have found that gut microbiota diversity metrics, composition, functional capacity, and metabolites can have a dose-dependent response to certain chemical toxicants, for example, tetracycline^79^, nanoplastics^80^, and BPA/BPA alternatives^81^. While the effects we measured on gut microbiome diversity are significant, the impact of BaP exposure concentration appears to be somewhat stochastic, evidenced in the way that we observed extensive variation among microbiome samples collected from fish exposed to the same concentration of BaP. This is consistent with research that characterized host response to BaP exposure; BaP is a precursor metabolite that is biotransformed into toxic compounds by the liver, so the host response itself is also highly variable^16,39^. That said, we do find that the ASVs observed in the gut are able to predict exposure concentration much better than random chance, which supports the notion that BaP exposure results in ecological selection for specific functional groups of microbiota in the gut. Collectively, these observations indicate that assembly of the zebrafish gut is a relatively stochastic process, likely influenced by random sampling of the meta-community in the embryonic media and priority effects^66,82,83^, and that BaP exposure increases selective pressure for particular community assemblages with high interindividual variability, as is common in other xenobiotic studies^23^.

Given that the gut microbiome can impact brain functioning through the gut-brain axis^29–37^, we sought to determine if BaP-induced variation in the gut microbiome associates with larval photomotor response (LPR). Prior work demonstrated that the gut microbiome contributes to vertebrate behavior development^30,31,66,67,76–78^, and that specific gut isolates from the zebrafish gut can modulate larval photomotor response^34^. However, it is unclear whether exposure-related changes to gut microbiome assembly associate with alterations to behavioral development. Several lines of evidence in our study support the hypothesis that, at least in the case of embryonic BaP exposure, environmental chemicals induce perturbations to the gut microbiome that link to exposure-induced variation in behavior development. First, we found that both the alpha- and beta-diversity of the gut microbiome explains variation in LPR, in terms of light and dark cycle swimming activity. Additionally, a random forest analysis found that the relative abundance distributions of specific subsets of taxa in the gut predict LPR measures in zebrafish exposed to BaP. These taxa notably include members of the Shewanellaceae, members of which have been shown to metabolize BaP and influence behavior in mice^68–70^. Collectively, these results indicate that the larval zebrafish gut microbiome links to zebrafish behavior. We then sought to determine if the effect of BaP exposure on the microbiome explains exposure-associated variation in fish behavior. Our analyses find that the combined interaction between microbiome Shannon entropy and BaP exposure concentration explains variation in dark cycle LPR activity. Additionally, while we observed no interaction effects between BaP exposure concentration and behavior on microbiome beta-diversity, we resolved several taxa that manifest different associations with behavior measures as a function of BaP exposure concentration. These observations included members of the Lachnospiraceae, which have been linked to BaP degradation as well as mammalian behavior^73^. Taken together, these results indicate that the gut microbiome not only associates with larval behavior, but also does so in a dose-dependent manner and suggests that exposure-induced perturbations to the gut microbiome may be a mechanism through which BaP elicits its neuroactive effects.

Given these observations, we next sought to determine whether the gut microbiome dictates the larval photomotor response to BaP exposure through a germ-free zebrafish experiment. Prior work has demonstrated that germ-free fish are hyperactive relative to conventionally reared zebrafish^48,56^, a finding that is replicated in our study. We subsequently sought to determine whether the colonization state of the gut microbiome determines BaP-driven developmental neurotoxicity. Our results indicate that the gut microbiome plays a role in determining the effect of embryonic BaP exposure on behavioral development in zebrafish. Specifically, the presence of the gut microbiome modulated the impact of BaP on light cycle LPR. Additionally, microbiome state elicited an additive effect alongside BaP exposure concentration on dark cycle LPR phenotype. These observations hold implications for our understanding of how BaP elicits its neurotoxic effects and suggests that the presence of the microbiome, if not also its compositional state, is at least one major factor defining toxicity. However, it remains unclear how these effects manifest. Recent research suggests that vertebrate receptors for environmental chemical toxicants like BaP that have an effect on neurodevelopment are also associated with gut microbial composition^19,26,27^ and can bind microbial metabolites^26^, supporting the idea that gut microbiota and their hosts have evolved methods of responding to toxicant exposure in concert. The present study and others have also established a strong connection between xenobiotic exposure and dysbiosis^14,16,20,22,47,81^, and gut dysbiosis is often associated with atypical behavior via the gut-brain axis^33,36,37,84–86^, providing yet another link between host neurophysiology, toxicant exposure, and gut microbiota. The results presented here reveal new factors to consider in the effort to define the cellular mechanisms through which BaP impacts neurodevelopment, namely microbiome metabolism of BaP as well as BaP-induced gut dysbiosis.

Overall, observations derived from this study are consistent with the hypothesis that environmental neurotoxicants interact with and impact the gut microbiome in ways that influence developmental neurotoxicity. Future research can build upon these observations to improve the strength of this hypothesis in several ways. We identified taxa perturbed by BaP, and future work should seek to clarify the specific role these taxa may play in defining zebrafish neurodevelopment. Studies in mammal models as well as human populations should also consider whether BaP exposure links to perturbed gut microbiome assembly and whether these effects drive neurodevelopmental disorders. Additionally, while our investigation reveals the impact of BaP on microbiome assembly through early life, less is known about how embryonic exposure to BaP impacts the successional development of the microbiome throughout lifespan. Given that embryonic BaP exposure drives neurodegenerative phenotypes in adult zebrafish, which is a phenotype that is transgenerationally inherited^3,56,87^, it would be valuable to determine if the entanglement our study has uncovered with respect to exposure, the gut microbiome, and behavior also manifests later in life and even across generations. Finally, it is critical that we ultimately define the molecular mechanisms the microbiome utilizes to resist the effects of BaP as well as those used to mediate BaP neurotoxicity in the effort to combat the toxic developmental effects of this ubiquitous environmental pollutant.

BaP is a ubiquitous environmental chemical toxicant that is impossible to escape in modern, industrialized countries^38,39^. Past studies have focused on the effect of differential exposures to host physiology, in model organisms and humans, including but not limited to behavioral responses^38,39,42,43,46–49,56,67^. Furthermore, BaP is a precursor molecule to toxic compounds with myriad endpoints in vertebrate hosts^16,39^, so defining a mechanism for how BaP affects the host is challenging. This range in response could be in part attributed to the gut microbiome, which in recent years has been implicated as a possible mechanism in both chemical toxicant exposure responses and behavior (via the gut-brain axis)^4,13,14,16–18,22,23,29–34,34–37,66,67,77,81,84–86,88^, as well as a reason behind the high inter-individual variability in xenobiotic response^23^. We found evidence not only that BaP is associated with measurable shifts in microbial assembly in early life, but also that the gut microbiome impacts the developmental neurotoxicity of BaP in zebrafish. Our work clarifies growing concerns surrounding BaP-induced neurotoxicity and suggests that continued exploration of the gut microbiome’s role in this toxicity may reveal novel strategies of mitigating adverse outcomes of exposure.

## Methods

### Experimental

#### The microbiome as an underlying variable in BaP’s impact on behavioral development

A workflow of our experiments is visualized in Fig. 1. All experiments were done with an approved IACUC protocol through Oregon State University (number 2020–0136) and followed guidelines for ethical treatment of animals. We gathered 864 5-D zebrafish^89^ embryos from a single clutch. Two thirds of these embryos underwent germ-free derivation as per a standard protocol^90^. In brief, the embryos are collected in embryo medium (EM) with antibiotics (100 µg/mL ampicillin, 250 ng/mL amphotericin B, 10 µg/mL gentamicin, 1 µg/mL tetracycline, and 1 µg/mL chloramphenicol). EM consists of 15 mM NaCl, 0.5 mM KCl, 1 mM CaCl2, 1 mM MgSO4, 0.15 mM KH2PO4, 0.05 mM Na2HPO4, and 0.7 mM NaHCO3 buffered with 1 M NaOH to pH 7.2^75^. Embryos are then washed briefly in a 0.1% PVP-I solution, rinsed three times with filter-sterilized EM, washed in a 0.003% bleach solution, and rinsed with filtered EM three times again. Each embryo was placed in a well of a 96-well plate containing 100 µL of EM. For the germ-free treatment group, this EM was filter-sterilized. For the conventionalized treatment group, which also underwent germ-free derivation, and the conventional treatment group, this EM was not filter-sterilized. All plates were then covered with a silicon pressure activated seal that permits the passage of gasses, but not water, bacteria, or particulates. For two rows of each plate (24 wells per plate) we included in the EM a 0 (0.1% DMSO vehicle), 1, 5, or 10 µM concentration of benzo-[*a*]-pyrene (BaP) (CAS Registry Number 50–32-8). The plates were stored for the first 24 h in the dark at 28 °C and subsequently on a 14/10 light–dark cycle also at 28 °C. Embryonic behavioral development was assessed at 1 day post fertilization (dpf) using the embryonic photomotor response (EPR) assay, and larval behavioral development was assessed at 5 dpf using the larval photomotor response (LPR) assay^48,56^. Only the last 3 epoch of the LPR assay was used for analysis. At both time points, embryos and larvae were visually inspected for deformities and mortality.

#### Effects of BaP on the gut microbiome and its link to behavior

We gathered 576 zebrafish embryos from a single clutch and placed each one in a well of a 96-well plate following the same rearing conditions as described above for conventional embryos. These embryos also underwent the same BaP exposure design and behavioral assessments as detailed above. At 9 dpf, all surviving larvae underwent intestinal dissection following all appropriate IACUC guidelines, including anesthetization by immersion in an ice bath for 20 min prior to dissection. 9 dpf is the earliest age at which fish could reasonably have their intestines removed, from a technical standpoint. Dissected intestines were placed directly in DNA extraction tubes provided by the DNeasy PowerSoil DNA Extraction kits (Qiagen, Hilden, Germany), which contain a DNA-stabilizing buffer, and were then flash frozen in liquid nitrogen. Samples were stored at -80 °C prior to DNA extraction.

### DNA extraction and sequencing

Extraction was conducted using the DNeasy PowerSoil DNA Extraction kit and sequencing libraries were prepared using EMP PCR protocol and primers in triplicate to amplify the V4 region of the 16S rRNA gene. Libraries were submitted to the Center for Quantitative Life Sciences at Oregon State University for sequencing on the Illumina MiSeq high-throughput sequencer (San Diego, California, USA).

### Sequence QC and processing

Raw reads were filtered for quality, merged, and assigned to amplicon sequence variants (ASVs) using the dada2 R package ^91^ as implemented in our lab’s pipeline (https://github.com/kstagaman/sharpton-lab-dada2-pipeline). ASVs were assigned a taxonomy down to the genus level using dada2 and the Silva database (version 138.1)^92^. For phylogenetic analysis, ASVs were aligned multiply using the NAST algorithm as implemented in mothur^93^ along with guide sequences chosen from the Silva database to maximally represent 16S diversity^17^. A phylogeny of the microbiome was inferred using FastTree2^94^, an approximately-maximum-likelihood method.

### Diversity metrics

All analyses were conducted at the ASV level unless otherwise noted. We estimated five alpha-diversity metrics for each larval zebrafish sample: richness (observed ASVs), Chao1^95^, Shannon^96^, Simpson^97^, and phylogenetic diversity (Faith’s PD^98^). We also estimated beta-diversity between each pair of larval zebrafish samples using six metrics. These included two phylogeny-agnostic metrics—Sørensen (abundance-unweighted)^99^ and Canberra (abundance-ranked)^100^—as well as two phylogenetic metrics: unweighted UniFrac and weighted UniFrac^101^.

### Behavior data

For EPR and LPR movement data, we calculated the areas under the curve (AUCs) from the movement data for each zebrafish sample as per previous studies, via a custom camera for EPR and a ZebraBox for LPR^64,102,103^. For the LPR results, there are four epochs of light and dark cycles. In analyzing these data, the initial epoch (epoch 0 [E0]) is the acclimation period and disregarded from data analysis. We calculated AUCs for the remaining epochs on a per epoch (E1–E3) per cycle (light vs dark) basis as well as summing across all epochs (i.e., a total AUC for the dark cycle and a total AUC for the light cycle). These total AUCs (µm × min [difference in movement multiplied by difference in timepoint]) were utilized for the statistical analyses.

### Statistical analyses

For analysis of single response variables (e.g., AUC, alpha-diversity), we utilized Gaussian linear mixed-effects models with plate ID as a random factor. The only exception to this was for testing the effects of microbiome treatment (conventional vs. conventionalized vs. germ-free) on larval behavior as, due to methodological constraints, plate ID and microbiome treatment had to be conflated. For these tests, we used Gaussian generalized linear regression models. For beta-diversity analyses, where a distance matrix is the response variable, we tested associations using PERMANOVA models conditioned on plate ID (i.e., the model accounts for the variance explained by plate ID first, and then tests the effects of other model predictors on the residuals). All linear models with multiple predictors were optimized by comparing increasingly complex models to a base model with one predictor using an ANOVA test. PERMANOVA models with multiple predictors were optimized using the function ordistep from the vegan package which adds and drops individual terms to minimize Aikake’s Information Criterion (AIC). These optimized models were then used for testing the significance of associations between response variables and predictors with a significance threshold of 0.05. We implemented random forest models using the ranger package^104^ and chose hyper-parameters for the models using the caret package^105^. We utilized the random forest models to determine how well taxon abundances could predict BaP exposure and LPR AUCs. For all models, we compared two data sets: ASV-only and aggregated (wherein we included ASV abundances as well as summed abundances for all taxonomic levels from genus to phylum). For predicting BaP exposure, we utilized both classification and regression random forest models, which were congruent in terms of outcomes, so we report here the classification model. For predicting LPR AUCs, we only utilized multivariate regression models.

### Supplementary Information

Supplementary Information.

## Data Availability

Raw sequences are available in the NCBI short read archive under the BioProject PRJNA887407 (https://www.ncbi.nlm.nih.gov/bioproject/ PRJNA887407). Code for analyses can be accessed at https://github.com/aalexiev/BaP_16S.

## References

[1] Plomin R (1986). Development, Genetics, and Psychology.

[2] Plomin R (1994). Genetics and experience. Curr. Opin. Psychiatry.

[3] Gao D, Wu M, Wang C, Wang Y, Zuo Z (2015). Chronic exposure to low benzo[a]pyrene level causes neurodegenerative disease-like syndromes in zebrafish (Danio rerio). Aquat Toxicol.

[4] Zheng D, Ba F, Bi G, Guo Y, Gao Y, Li W (2020). The sharp rise of neurological disorders associated with recreational nitrous oxide use in China: A single-center experience and a brief review of Chinese literature. J Neurol.

[5] Reynolds A, Laurie C, Lee Mosley R, Gendelman HE (2007). Oxidative Stress and the Pathogenesis of Neurodegenerative Disorders. International Review of Neurobiology.

[6] Nabi M, Tabassum N (2022). Role of environmental toxicants on neurodegenerative disorders. Front. Toxicol..

[7] Barlow BK, Cory-Slechta DA, Richfield EK, Thiruchelvam M (2007). The gestational environment and Parkinson’s disease: Evidence for neurodevelopmental origins of a neurodegenerative disorder. Reprod. Toxicol..

[8] Aschner M, Costa LG (2015). Environmental Factors in Neurodevelopmental and Neurodegenerative Disorders.

[9] Devi S, Kumar V, Singh SK, Dubey AK, Kim J-J (2021). Flavonoids: Potential candidates for the treatment of neurodegenerative disorders. Biomedicines.

[10] Migliore L, Coppedè F (2009). Environmental-induced oxidative stress in neurodegenerative disorders and aging. Mutat. Res./Genet. Toxicol. Environ. Mutagen..

[11] Spencer PS, Ludolph AC, Kisby GE (1992). Are human neurodegenerative disorders linked to environmental chemicals with excitotoxic properties. Ann. N. Y. Acad. Sci..

[12] Rodrigues JA, Narasimhamurthy RK, Joshi MB, Dsouza HS, Mumbrekar KD (2022). Pesticides exposure-induced changes in brain metabolome: Implications in the pathogenesis of neurodegenerative disorders. Neurotox. Res..

[13] Sutherland VL, McQueen CA, Mendrick D, Gulezian D, Cerniglia C, Foley S (2020). The gut microbiome and xenobiotics: Identifying knowledge gaps. Toxicol. Sci..

[14] Claus SP, Guillou H, Ellero-Simatos S (2016). The gut microbiota: A major player in the toxicity of environmental pollutants?. Npj Biofilms Microb..

[15] Lu K, Abo RP, Schlieper KA, Graffam ME, Levine S, Wishnok JS (2014). Arsenic exposure perturbs the gut microbiome and its metabolic profile in mice: An integrated metagenomics and metabolomics analysis. Environ. Health Perspect..

[16] Bertotto LB, Catron TR, Tal T (2020). Exploring interactions between xenobiotics, microbiota, and neurotoxicity in zebrafish. NeuroToxicol..

[17] Sharpton TJ, Stagaman K, Sieler MJ, Arnold HK, Davis EW (2021). Phylogenetic integration reveals the zebrafish core microbiome and its sensitivity to environmental exposures. Toxics.

[18] Tu P, Chi L, Bodnar W, Zhang Z, Gao B, Bian X (2020). Gut microbiome toxicity: Connecting the environment and gut microbiome-associated diseases. Toxics.

[19] Dong F, Perdew GH (2020). The aryl hydrocarbon receptor as a mediator of host-microbiota interplay. Gut Microbes.

[20] Catron TR, Gaballah S, Tal T (2019). Using zebrafish to investigate interactions between xenobiotics and microbiota. Curr. Pharmacol. Rep..

[21] Weitekamp CA, Phelps D, Swank A, McCord J, Sobus JR, Catron T (2019). Triclosan-selected host-associated microbiota perform xenobiotic biotransformations in larval zebrafish. Toxicol. Sci..

[22] Chi L, Tu P, Ru H, Lu K (2021). Studies of xenobiotic-induced gut microbiota dysbiosis: from correlation to mechanisms. Gut Microbes.

[23] Maurice CF, Haiser HJ, Turnbaugh PJ (2013). Xenobiotics shape the physiology and gene expression of the active human gut microbiome. Cell.

[24] Claus SP, Ellero SL, Berger B, Krause L, Bruttin A, Molina J (2011). Colonization-induced host-gut microbial metabolic interaction. mBio.

[25] Claus SP, Tsang TM, Wang Y, Cloarec O, Skordi E, Martin F-P (2008). Systemic multicompartmental effects of the gut microbiome on mouse metabolic phenotypes. Mol. Syst. Biol..

[26] Dong F, Hao F, Murray IA, Smith PB, Koo I, Tindall AM (2020). Intestinal microbiota-derived tryptophan metabolites are predictive of Ah receptor activity. Gut Microbes.

[27] Zhang L, Nichols RG, Correll J, Murray IA, Tanaka N, Smith PB (2015). Persistent organic pollutants modify gut microbiota-host metabolic homeostasis in mice through aryl hydrocarbon receptor activation. Environ. Health Perspect..

[28] Breton J, Massart S, Vandamme P, De Brandt E, Pot B, Foligné B (2013). Ecotoxicology inside the gut: Impact of heavy metals on the mouse microbiome. BMC Pharmacol. Toxicol..

[29] Cryan JF, O’Mahony SM (2011). The microbiome-gut-brain axis: from bowel to behavior. Neurogastroenterol. Motil..

[30] Cryan JF, O’Riordan KJ, Cowan CSM, Sandhu KV, Bastiaanssen TFS, Boehme M (2019). The microbiota-gut-brain axis. Physiol. Rev..

[31] Cussotto S, Sandhu KV, Dinan TG, Cryan JF (2018). The neuroendocrinology of the microbiota-gut-brain axis: a behavioural perspective. Front. Neuroendocrinol..

[32] Lyte M (2013). Microbial endocrinology in the microbiome-gut-brain axis: How bacterial production and utilization of neurochemicals influence behavior. PLOS Pathog..

[33] Hsiao EY, McBride SW, Hsien S, Sharon G, Hyde ER, McCue T (2013). Microbiota modulate behavioral and physiological abnormalities associated with neurodevelopmental disorders. Cell.

[34] Phelps D, Brinkman NE, Keely SP, Anneken EM, Catron TR, Betancourt D (2017). Microbial colonization is required for normal neurobehavioral development in zebrafish. Sci. Rep..

[35] Tognini P (2017). Gut microbiota: A potential regulator of neurodevelopment. Front. Cell Neurosci..

[36] Sharon G, Sampson TR, Geschwind DH, Mazmanian SK (2016). The central nervous system and the gut microbiome. Cell.

[37] Flight MH (2014). The gut–microbiome–brain connection. Nat. Rev. Neurosci..

[38] Gelboin HV (1980). Benzo[alpha]pyrene metabolism, activation and carcinogenesis: role and regulation of mixed-function oxidases and related enzymes. Physiol. Rev..

[39] Bukowska B, Mokra K, Michałowicz J (2022). Benzo[a]pyrene—environmental occurrence, human exposure, and mechanisms of toxicity. Int. J. Mol. Sci..

[40] Kazerouni N, Sinha R, Hsu CH, Greenberg A, Rothman N (2001). Analysis of 200 food items for benzo[a]pyrene and estimation of its intake in an epidemiologic study. Food Chem. Toxicol..

[41] Méndez García M, García de Llasera MP (2021). A review on the enzymes and metabolites identified by mass spectrometry from bacteria and microalgae involved in the degradation of high molecular weight PAHs. Sci. Total Environ..

[42] Arnould JP, Verhoest P, Bach V, Libert JP, Belegaud J (1997). Detection of benzo[a]pyrene-DNA adducts in human placenta and umbilical cord blood. Hum. Exp. Toxicol..

[43] Perera FP, Tang D, Wang S, Vishnevetsky J, Zhang B, Diaz D (2012). Prenatal polycyclic aromatic hydrocarbon (PAH) exposure and child behavior at age 6–7 years. Environ. Health Perspect..

[44] Tian S, Pan L, Sun X (2013). An investigation of endocrine disrupting effects and toxic mechanisms modulated by benzo[a]pyrene in female scallop Chlamys farreri. Aquat. Toxicol..

[45] Borek-Dohalska L, Klusonova Z, Holecova J, Martinkova M, Barta F, Dracinska H (2016). Exposure of rats to exogenous endocrine disruptors 17alpha-ethinylestradiol and benzo(a)pyrene and an estrogenic hormone estradiol induces expression of cytochromes P450 involved in their metabolism. Neuro Endocrinol. Lett..

[46] He C, Wang C, Zhou Y, Li J, Zuo Z (2012). Embryonic exposure to benzo(a)pyrene influences neural development and function in rockfish (*Sebastiscus marmoratus*). Neurotoxicology.

[47] Das SK, Patel B, Patri M (2016). Neurotoxic effect of benzo[a]pyrene and Its possible association with 6-hydroxydopamine induced neurobehavioral changes during early adolescence period in rats. J. Toxicol..

[48] Knecht AL, Truong L, Simonich MT, Tanguay RL (2017). Developmental benzo[a]pyrene (B[a]P) exposure impacts larval behavior and impairs adult learning in zebrafish. Neurotoxicol. Teratol..

[49] Zhang W, Tian F, Zheng J, Li S, Qiang M (2016). Chronic administration of benzo(a)pyrene induces memory impairment and anxiety-like behavior and increases of NR2B dna methylation. PLoS One.

[50] Mohanty R, Das SK, Singh NR, Patri M (2016). *Withania somnifera* leaf extract ameliorates benzo[a]pyrene-induced behavioral and neuromorphological alterations by improving brain antioxidant status in zebrafish (*Danio rerio*). Zebrafish.

[51] Defois C, Ratel J, Denis S, Batut B, Beugnot R, Peyretaillade E (2017). Environmental pollutant benzo[a]pyrene impacts the volatile metabolome and transcriptome of the human gut microbiota. Front. Microbiol..

[52] Ribière C, Peyret P, Parisot N, Darcha C, Déchelotte PJ, Barnich N (2016). Oral exposure to environmental pollutant benzo[a]pyrene impacts the intestinal epithelium and induces gut microbial shifts in murine model. Sci. Rep..

[53] Wood AW, Wislocki PG, Chang RL, Levin W, Lu AY, Yagi J (1976). Mutagenicity and cytotoxicity of benzo(a)pyrene benzo-ring epoxides. Cancer Res..

[54] Wislocki PG, Wood AW, Chang RL, Levin W, Yagi H, Hernandez O (1976). Mutagenicity and cytotoxicity of benzo(a)pyrene arene oxides, phenols, quinones, and dihydrodiols in bacterial and mammalian cells. Cancer Res..

[55] Seo J-S, Keum Y-S, Li QX (2009). Bacterial degradation of aromatic compounds. Int. J. Environ. Res. Public Health.

[56] Knecht AL, Truong L, Marvel SW, Reif DM, Garcia A, Lu C (2017). Transgenerational inheritance of neurobehavioral and physiological deficits from developmental exposure to benzo[a]pyrene in zebrafish. Toxicol. Appl. Pharmacol..

[57] Phillips DH (1983). Fifty years of benzo(a)pyrene. Nature.

[58] Leshem A, Segal E, Elinav E (2020). The gut microbiome and individual-specific responses to diet. mSystems.

[59] Gacesa R, Kurilshikov A, Vila AV, Sinha T, Klaassen M, a. Y, Bolte LA,  (2020). The Dutch microbiome project defines factors that shape the healthy gut microbiome. BioRxiv.

[60] MacPhail RC, Brooks J, Hunter DL, Padnos B, Irons TD, Padilla S (2009). Locomotion in larval zebrafish: Influence of time of day, lighting and ethanol. Neuro Toxicol..

[61] Truong L, Saili KS, Miller JM, Hutchison JE, Tanguay RL (2012). Comparative biochemistry and physiology, Part C persistent adult zebrafish behavioral deficits results from acute embryonic exposure to gold nanoparticles. Comp. Biochem. Physiol. Part C.

[62] Noyes PD, Haggard DE, Gonnerman GD, Tanguay RL (2015). Advanced morphological—behavioral test platform reveals neurodevelopmental defects in embryonic zebrafish exposed to comprehensive suite of halogenated and organophosphate flame retardants. Toxicolog. Sci..

[63] Crosby EB, Bailey JM, Oliveri AN, Levin ED (2015). Neurobehavioral impairments caused by developmental imidacloprid exposure in zebrafish. Neurotoxicol. Teratol..

[64] Reif DM, Truong L, Mandrell D, Marvel S, Zhang G, Tanguay RL (2016). High-throughput characterization of chemical-associated embryonic behavioral changes predicts teratogenic outcomes. Arch. Toxicol..

[65] Dasgupta S, Wang G, Simonich MT, Zhang T, Truong L, Liu H (2020). Impacts of high dose 3.5 GHz cellphone radiofrequency on zebrafish embryonic development. PLOS One.

[66] Burns AR, Stephens WZ, Stagaman K, Wong S, Rawls JF, Guillemin K (2016). Contribution of neutral processes to the assembly of gut microbial communities in the zebrafish over host development. ISME J..

[67] Stephens WZ, Burns AR, Stagaman K, Wong S, Rawls JF, Guillemin K (2016). The composition of the zebrafish intestinal microbial community varies across development. ISME J..

[68] Rathour R, Medhi K, Gupta J, Thakur IS (2021). Integrated approach of whole-genome analysis, toxicological evaluation and life cycle assessment for pyrene biodegradation by a psychrophilic strain, *Shewanella* sp. ISTPL2. Environ Pollut.

[69] Rathour R, Gupta J, Tyagi B, Kumari T, Thakur IS (2018). Biodegradation of pyrene in soil microcosm by *Shewanella* sp. ISTPL2, a psychrophilic, alkalophilic and halophilic bacterium. Bioresour. Technol. Rep..

[70] De Palma G, Lynch MDJ, Lu J, Dang VT, Deng Y, Jury J (2017). Transplantation of fecal microbiota from patients with irritable bowel syndrome alters gut function and behavior in recipient mice. Sci. Transl. Med..

[71] Roeselers G, Mittge EK, Stephens WZ, Parichy DM, Cavanaugh CM, Guillemin K (2011). Evidence for a core gut microbiota in the zebrafish. ISME J..

[72] Majeed A, Muhammad Z, Ullah R, Ullah K, Ali H, Inayat N, Naeem M, Aftab T, Ali Ansari A, Gill SS, Macovei A (2022). Chapter 18 - Plant growth-promoting rhizobacteria as bioremediators of polluted agricultural soils: Challenges and prospects. Hazardous and Trace Materials in Soil and Plants.

[73] Vacca M, Celano G, Calabrese FM, Portincasa P, Gobbetti M, De Angelis M (2020). The controversial role of human gut lachnospiraceae. Microorganisms.

[74] Li H-H, Chang S-C (2022). Bioremediation of decabromodiphenyl ether or benzo(a)pyrene-contaminated rice-paddy soil. J. Soils Sedim..

[75] Westerfield M (2007). The zebrafish book: A guide for the laboratory use of zebrafish (Danio rerio).

[76] Martino C, Dilmore AH, Burcham ZM, Metcalf JL, Jeste D, Knight R (2022). Microbiota succession throughout life from the cradle to the grave. Nat. Rev. Microbiol..

[77] Ahmed H, Leyrolle Q, Koistinen V, Kärkkäinen O, Layé S, Delzenne N (2022). Microbiota-derived metabolites as drivers of gut–brain communication. Gut Microbes.

[78] Liang S, Wu X, Jin F (2018). Gut-brain psychology: Rethinking psychology from the microbiota–gut–brain axis. Front. Integr. Neurosci..

[79] Keerthisinghe TP, Wang F, Wang M, Yang Q, Li J, Yang J (2020). Long-term exposure to TET increases body weight of juvenile zebrafish as indicated in host metabolism and gut microbiome. Environ. Int..

[80] Teng M, Zhao X, Wang C, Wang C, White JC, Zhao W (2022). Polystyrene nanoplastics toxicity to zebrafish: Dysregulation of the brain–intestine–microbiota axis. ACS Nano.

[81] Catron TR, Keely SP, Brinkman NE, Zurlinden TJ, Wood CE, Wright JR (2019). Host developmental toxicity of BPA and BPA alternatives is inversely related to microbiota disruption in zebrafish. Toxicol. Sci..

[82] Sprockett D, Fukami T, Relman DA (2018). Role of priority effects in the early-life assembly of the gut microbiota. Nat. Rev. Gastroenterol. Hepatol..

[83] Debray R, Herbert RA, Jaffe AL, Crits-Christoph A, Power ME, Koskella B (2022). Priority effects in microbiome assembly. Nat. Rev. Microbiol..

[84] Willyard C (2021). How gut microbes could drive brain disorders. Nature.

[85] Oroojzadeh P, Bostanabad SY, Lotfi H (2022). Psychobiotics: The influence of gut microbiota on the gut-brain axis in neurological disorders. J. Mol. Neurosci..

[86] Carabotti M, Scirocco A, Maselli MA, Severi C (2015). The gut-brain axis: Interactions between enteric microbiota, central and enteric nervous systems. Ann. Gastroenterol..

[87] Gao D, Lin J, Ou K, Chen Y, Li H, Dai Q (2018). Embryonic exposure to benzo(a)pyrene inhibits reproductive capability in adult female zebrafish and correlation with DNA methylation. Environ. Pollut..

[88] Weitekamp CA, Kvasnicka A, Keely SP, Brinkman NE, Howey XM, Gaballah S (2021). Monoassociation with bacterial isolates reveals the role of colonization, community complexity and abundance on locomotor behavior in larval zebrafish. Anim. Microb..

[89] Kent ML, Buchner C, Watral VG, Sanders JL, Ladu J, Peterson TS (2011). Development and maintenance of a specific pathogen-free (SPF) zebrafish research facility for *Pseudoloma neurophilia*. Dis. Aquat. Organ..

[90] Melancon E, De La Torre Canny SG, Sichel S, Kelly M, Wiles TJ, Rawls JF (2017). Best practices for germ-free derivation and gnotobiotic zebrafish husbandry. Methods Cell Biol..

[91] Callahan BJ, McMurdie PJ, Rosen MJ, Han AW, Johnson AJA, Holmes SP (2016). DADA2: High-resolution sample inference from Illumina amplicon data. Nat. Methods.

[92] Quast C, Pruesse E, Yilmaz P, Gerken J, Schweer T, Yarza P (2013). The SILVA ribosomal RNA gene database project: Improved data processing and web-based tools. Nucleic Acids Res..

[93] Schloss PD, Westcott SL, Ryabin T, Hall JR, Hartmann M, Hollister EB (2009). Introducing mothur: open-source, platform-independent, community-supported software for describing and comparing microbial communities. Appl. Environ. Microbiol..

[94] Price MN, Dehal PS, Arkin AP (2010). FastTree 2–approximately maximum-likelihood trees for large alignments. PLoS One.

[95] Chao A, Shen T-J (2003). Nonparametric estimation of Shannon’s index of diversity when there are unseen species in sample. Environ. Ecol. Stat..

[96] Shannon CE (1948). A mathematical theory of communication. Bell Syst. Techn. J..

[97] Simpson EH (1949). Measurement of diversity. Nature.

[98] Faith DP (1992). Conservation evaluation and phylogenetic diversity. Biol. Conserv..

[99] Sørensen TJ (1948). A method of establishing groups of equal amplitude in plant sociology based on similarity of species content and its application to analyses of the vegetation on Danish commons.

[100] Lance GN, Williams WT (1967). Mixed-data classificatory programs I—agglomerative systems. Aust. Comput. J..

[101] Chen, J., Zhang, X. & Yang, L. GUniFrac: Generalized UniFrac distances and distance-based multivariate analysis of variance (2021).

[102] Zhang G, Truong L, Tanguay RL, Reif DM (2017). A new statistical approach to characterize chemical-elicited behavioral effects in high-throughput studies using zebrafish. PLOS ONE.

[103] Truong L, Reif DM, St Mary L, Geier MC, Truong HD, Tanguay RL (2014). Multidimensional in vivo hazard assessment using zebrafish. Toxicol Sci.

[104] Wright MN, Ziegler A (2017). ranger: A fast implementation of random forests for high dimensional data in C++ and R. J. Stat. Softw..

[105] Kuhn, M., Wing, J., Weston, S., Williams, A., Keefer, C., Engelhardt, A., *et al*. caret: Classification and regression training (2020).

