# Supplementary material for: The zebrafish gut microbiome influences benzo[a]pyrene developmental neurobehavioral toxicity
Source: Sci Rep. 2024 Jun 25;14:14618. doi: 10.1038/s41598-024-65610-3 (PMC11199668; doi:10.1038/s41598-024-65610-3)
Supplement: Supplementary file 1 — Supplementary Information. [file 41598_2024_65610_MOESM1_ESM.docx]

## **Supplemental Figures/Tables and Legends**


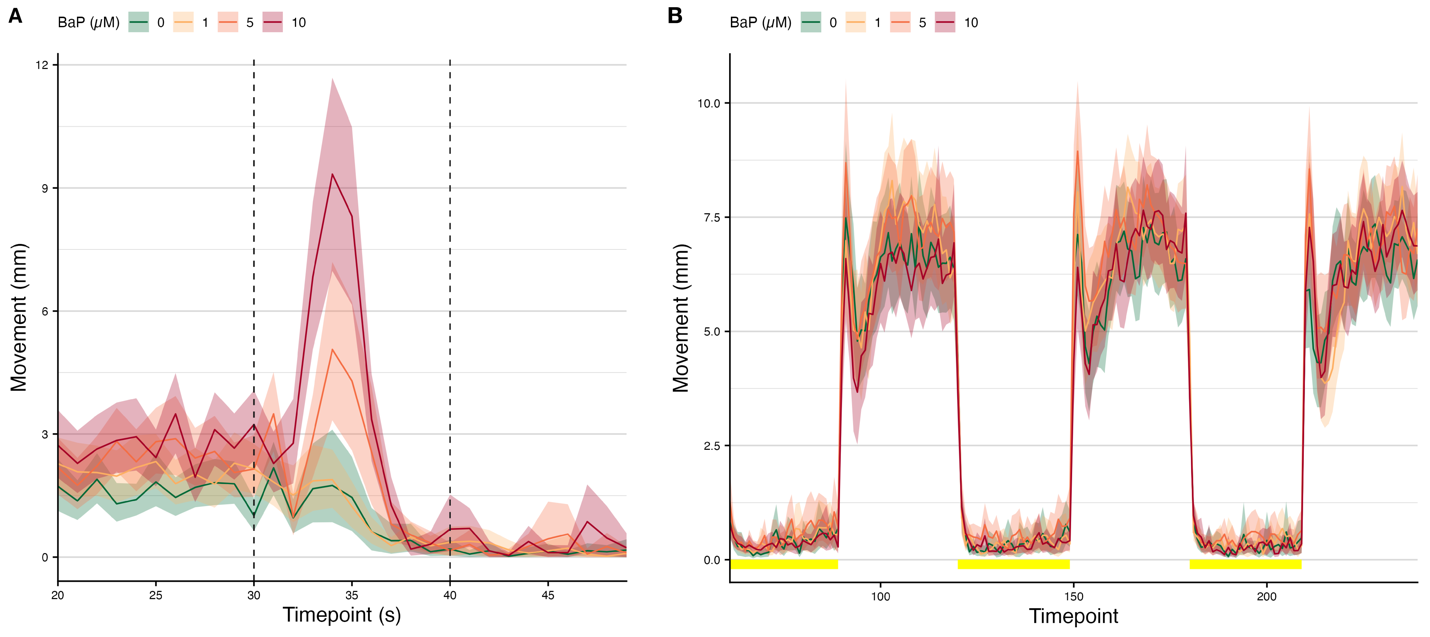
Supplemental Figure 1: BaP dosage effect on behavior metrics (A) EPR movement data. Colored lines indicate the mean movement (mm) for zebrafish embryos for each BaP exposure level (in µM). The shaded ribbons indicate 95% C.I.s for the movement means. The black dotted lines indicate the window of time on which later statistical analyses are based. (B) Colored lines indicate the mean movement (mm) for zebrafish embryos for each BaP exposure level (in μM). The shaded ribbons indicate 95% C.I.s for the movement means. The yellow segments near the x-axis indicate which time points fall within light cycles (the rest of the time points are in dark cycles).


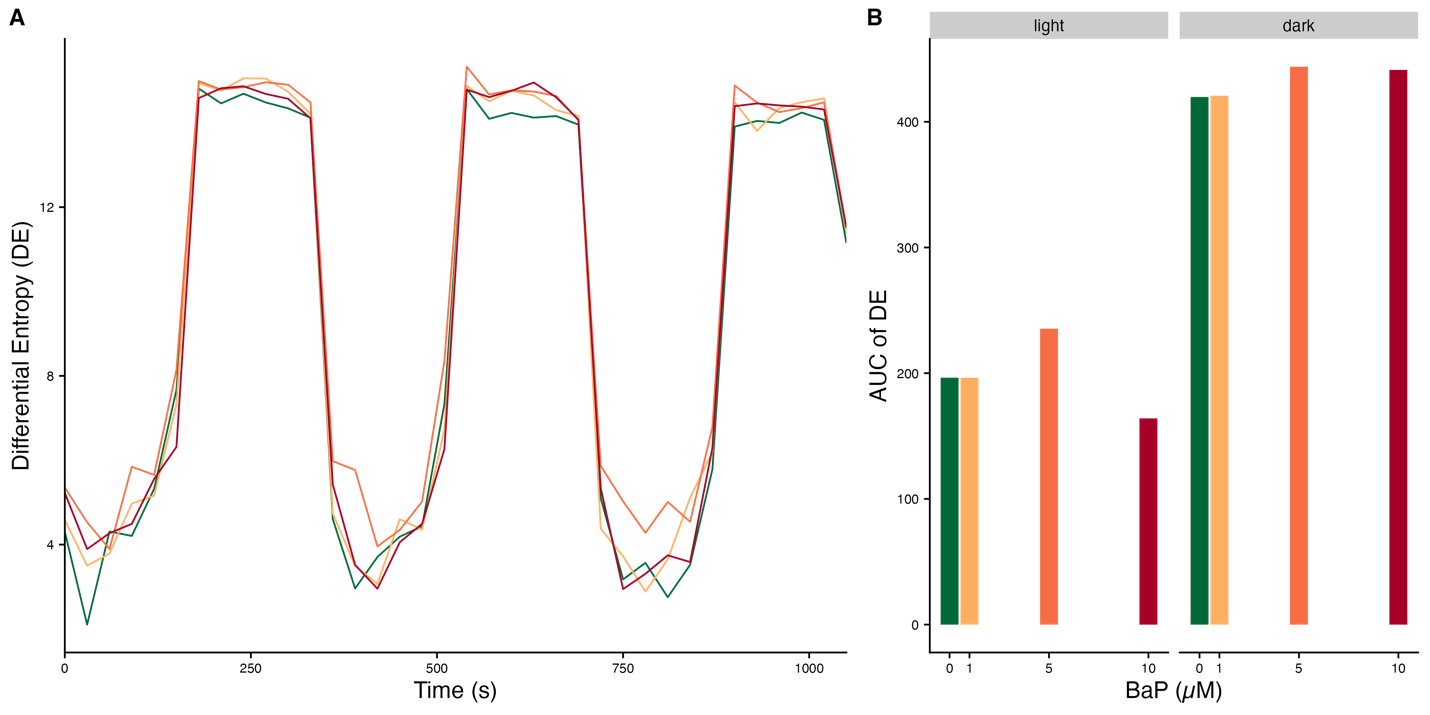
Supplemental Figure 2: Plots of the differential entropy (DE) calculated per BaP exposure per 96-well plate per LPR cycle (dark vs light). (A) DE over time for the LPR assay. (B) AUCs of the DE plots in panel A by light and dark cycles.


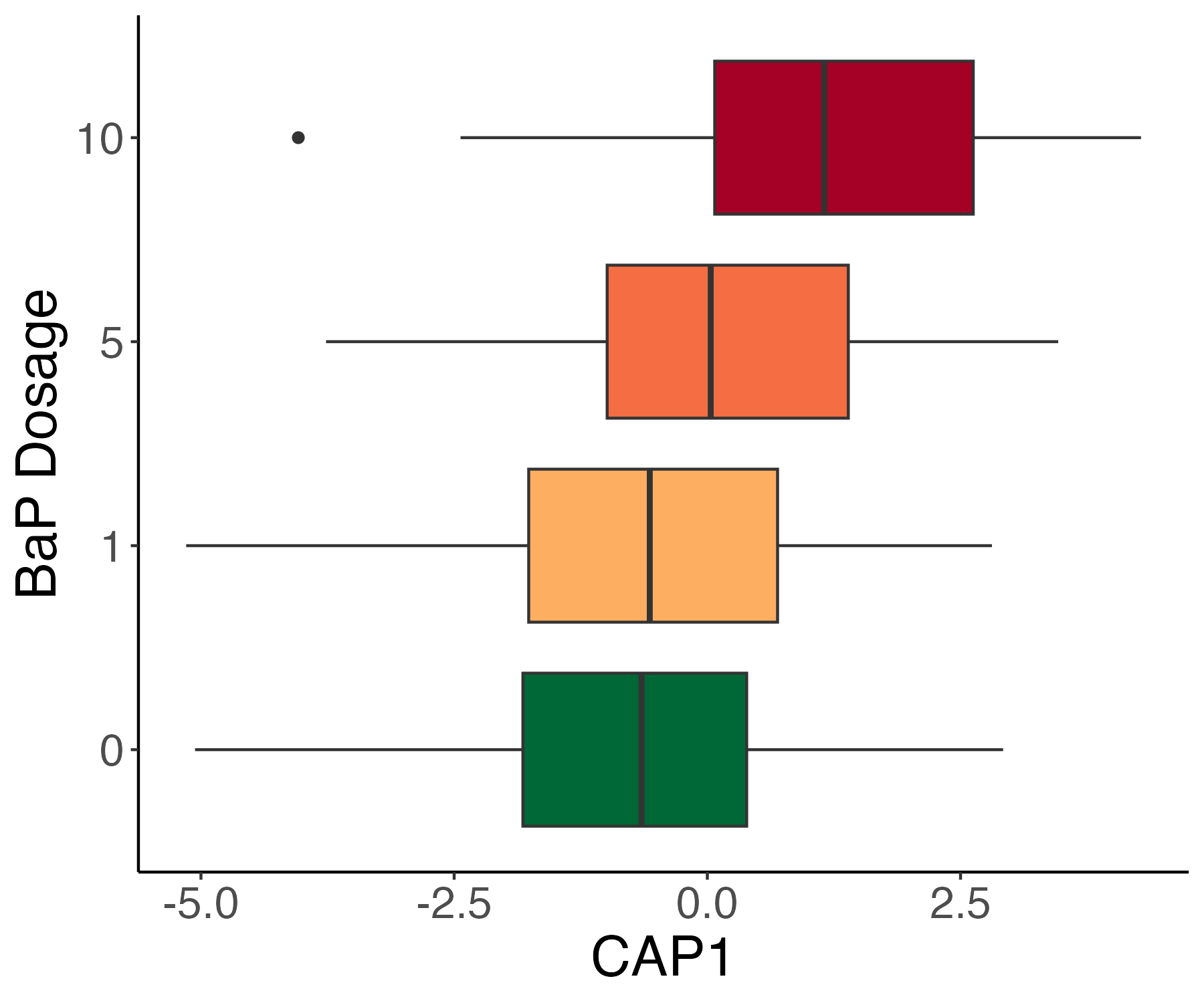


Supplemental Figure 3: A box and whisker plot of BaP dosage versus CAP1 position from the ordination in the panel B. The median of each box represents the median centroid location of the cluster on CAP1 for each BaP dosage from the ordination.


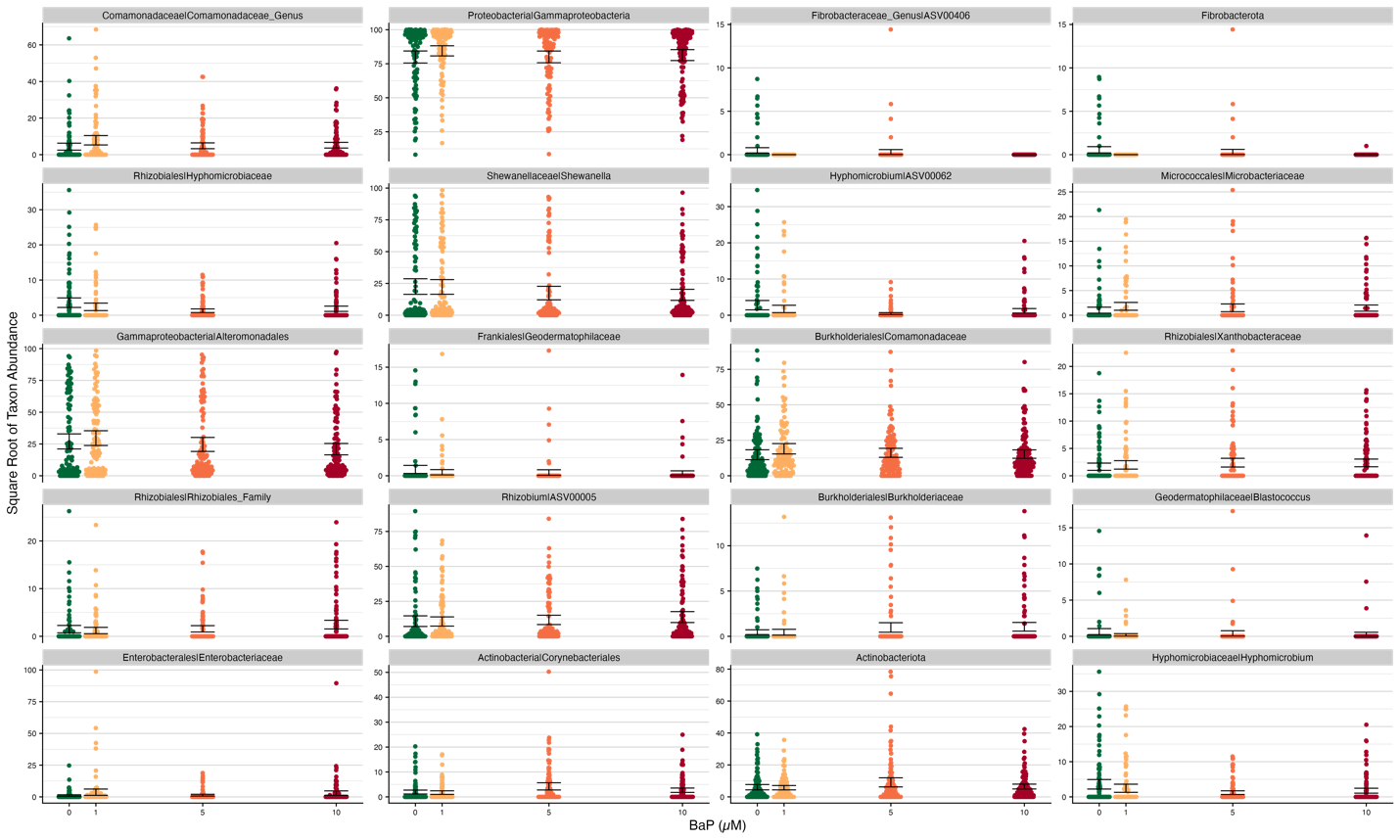
Supplemental Figure 4: Plots of the square root of abundances by BaP exposure for the top 20 significantly most important taxa for the random forest model (classification) predicting BaP exposure from taxon abundances.


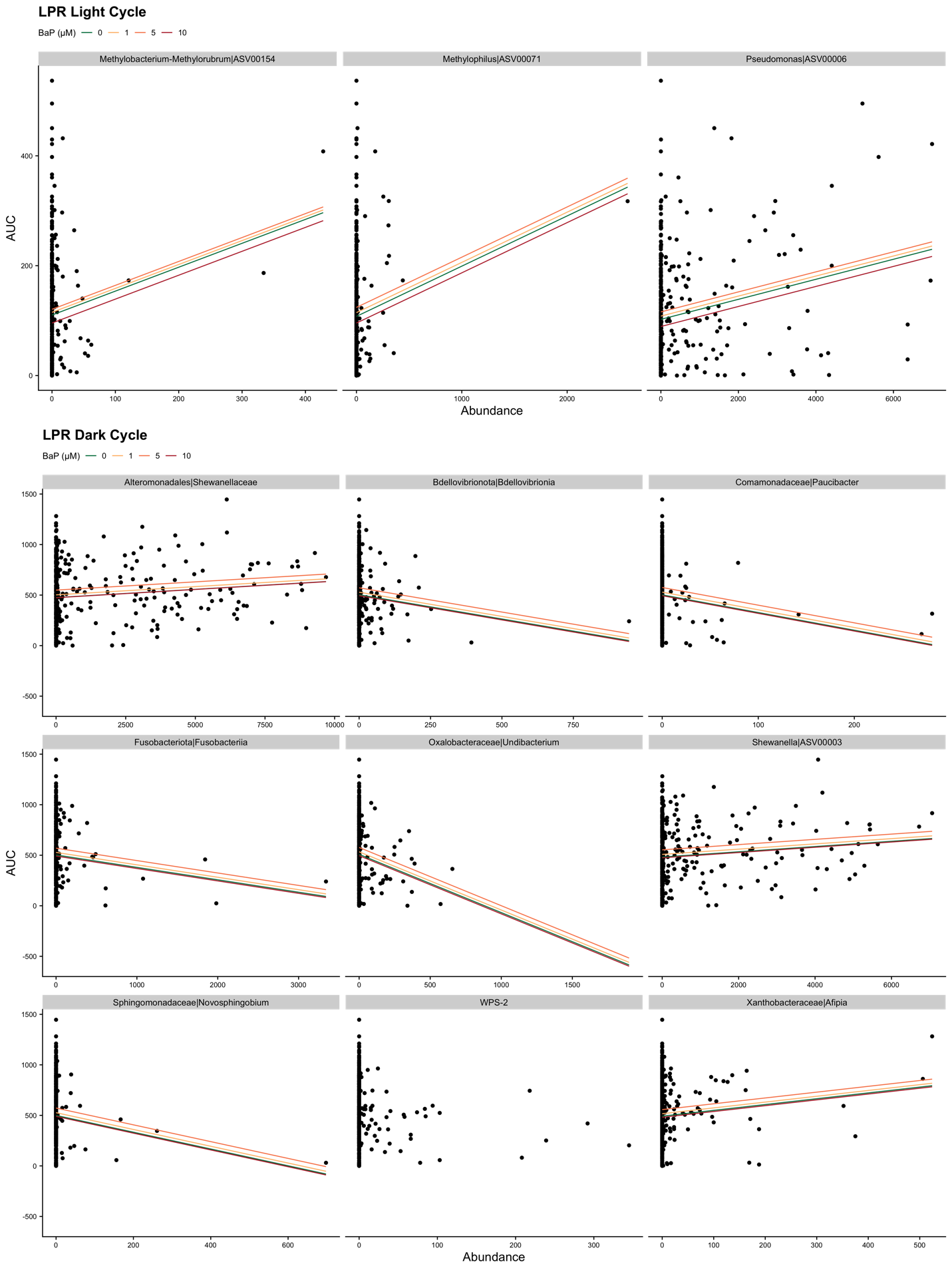
Supplemental Figure 5: Scatter plots of the relationship between individual taxon abundances and LPR light and dark cycle AUCs. In this figure, only statistically significant associations (*p* < 0.05) with no abundance-by-BaP interactions are shown. Lines represent estimated relationships from gaussian generalized linear models. Light cycle associated taxa are shown on the top row and dark cycle associated taxa are show in the three rows below. The colored lines represent each concentration of BaP in µM.


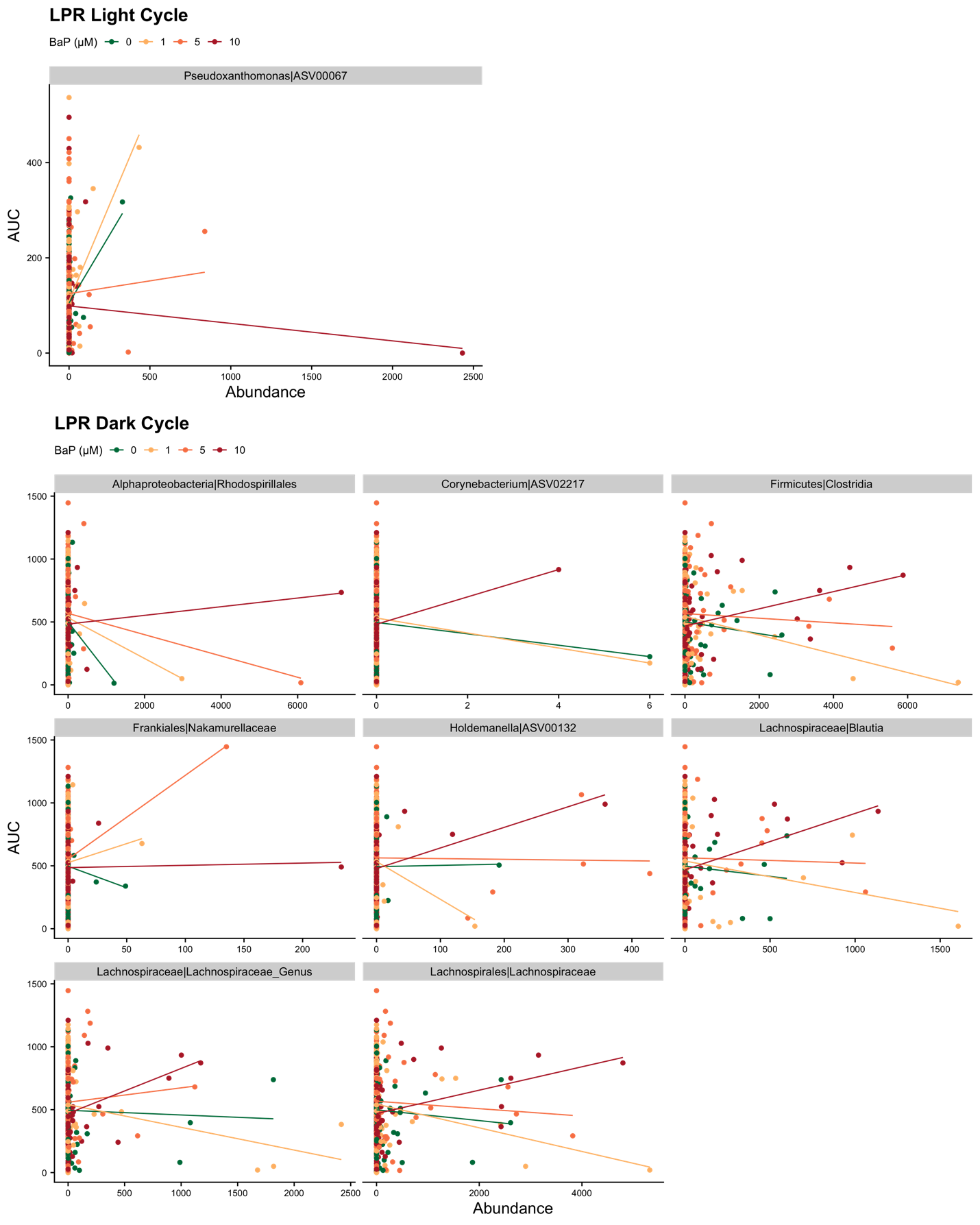
Supplemental Figure 6: Scatter plots of the relationship between individual taxon abundances and LPR light and dark cycle AUCs. In this figure, only statistically significant associations (*p* < 0.05) with abundance-by-BaP interactions are shown. Lines represent estimated relationships from gaussian generalized linear models. Points and lines are colored by BaP exposure level. Light cycle associated taxa are shown on the top row and dark cycle associated taxa are show in the three rows below.

Supplemental Table 1: Linear regression model testing the association between BaP exposure and EPR AUC values (embryonic activity).

| **Term** | **Estimate** | **Std.error** | **Statistic** | **P.value** | **Sig** |
| --- | --- | --- | --- | --- | --- |
| (Intercept) | 10.55 | 3.35 | 3.15 | 0.002 | * |
| BaP 1µM | 1.34 | 4.72 | 0.28 | 0.776 |  |
| BaP 5µM | 14.16 | 4.68 | 3.02 | 0.003 | * |
| BaP 10µM | 26.54 | 4.63 | 5.73 | 0 | * |

Supplemental Table 2: Linear regression models testing the association between BaP exposure and LPR AUC values (larval activity) by light and dark cycles.

| **Model** | **Cycle** | **Effect** | **Group** | **Term** | **Estimate** | **Std.error** | **Df** | **Statistic** | **P.value** | **Sig** |
| --- | --- | --- | --- | --- | --- | --- | --- | --- | --- | --- |
| 1st order | light | fixed |  | (Intercept) | 118.45 | 10.98 | 399 | 10.79 | 0 | * |
|  |  |  |  | BaP µM | -1.39 | 1.43 | 399 | -0.97 | 0.333 |  |
|  |  | ran_pars | SARL_plateID | sd_(Intercept) | 21.3 |  |  |  |  |  |
|  |  |  |  | cor_BaP_uM.(Intercept) | -0.36 |  |  |  |  |  |
|  |  |  |  | sd_BaP_uM | 2.05 |  |  |  |  |  |
|  |  |  | Residual | sd_Observation | 92.96 |  |  |  |  |  |
| 2nd order |  | fixed |  | (Intercept) | 110.39 | 11.46 | 398 | 9.63 | 0 | * |
|  |  |  |  | BaP µM | 7.75 | 4.74 | 398 | 1.64 | 0.102 |  |
|  |  |  |  | BaP µM^2 | -0.91 | 0.45 | 398 | -2.02 | 0.044 | * |
|  |  | ran_pars | SARL_plateID | sd_(Intercept) | 20.64 |  |  |  |  |  |
|  |  |  |  | cor_BaP_uM.(Intercept) | -0.3 |  |  |  |  |  |
|  |  |  |  | sd_BaP_uM | 2.03 |  |  |  |  |  |
|  |  |  | Residual | sd_Observation | 92.61 |  |  |  |  |  |
| 1st order | dark | fixed |  | (Intercept) | 525.63 | 25.87 | 399 | 20.32 | 0 | * |
|  |  |  |  | BaP µM | -1.64 | 3.72 | 399 | -0.44 | 0.66 |  |
|  |  | ran_pars | SARL_plateID | sd_(Intercept) | 39.47 |  |  |  |  |  |
|  |  |  |  | cor_BaP_uM.(Intercept) | 0.55 |  |  |  |  |  |
|  |  |  |  | sd_BaP_uM | 3 |  |  |  |  |  |
|  |  |  | Residual | sd_Observation | 281.09 |  |  |  |  |  |
| 2nd order |  | fixed |  | (Intercept) | 499.26 | 28.25 | 398 | 17.67 | 0 | * |
|  |  |  |  | BaP µM | 28.28 | 14.15 | 398 | 2 | 0.046 | * |
|  |  |  |  | BaP µM^2 | -2.96 | 1.35 | 398 | -2.19 | 0.029 | * |
|  |  | ran_pars | SARL_plateID | sd_(Intercept) | 38.6 |  |  |  |  |  |
|  |  |  |  | cor_BaP_uM.(Intercept) | 0.57 |  |  |  |  |  |
|  |  |  |  | sd_BaP_uM | 3.34 |  |  |  |  |  |
|  |  |  | Residual | sd_Observation | 279.72 |  |  |  |  |  |

Supplemental Table 3: Linear regression models testing the association between BaP exposure and larval microbiome alpha-diversity metrics.

| **Alpha.metric** | **Effect** | **Group** | **Term** | **Estimate** | **Std.error** | **Df** | **Statistic** | **P.value** | **Sig** |
| --- | --- | --- | --- | --- | --- | --- | --- | --- | --- |
| Chao1 | fixed |  | (Intercept) | 30.39 | 1.84 | 407 | 16.5 | 0 | * |
|  |  |  | BaP µM | 0.86 | 0.37 | 407 | 2.33 | 0.02 | * |
|  | ran_pars | SARL_plateID | sd_(Intercept) | 1.87 |  |  |  |  |  |
|  |  |  | cor_BaP_uM.(Intercept) | -0.91 |  |  |  |  |  |
|  |  |  | sd_BaP_uM | 0.55 |  |  |  |  |  |
|  |  | Residual | sd_Observation | 23.55 |  |  |  |  |  |
| Shannon | fixed |  | (Intercept) | 1.64 | 0.11 | 407 | 14.72 | 0 | * |
|  |  |  | BaP µM | 0.02 | 0.01 | 407 | 1.45 | 0.147 |  |
|  | ran_pars | SARL_plateID | sd_(Intercept) | 0.23 |  |  |  |  |  |
|  |  |  | cor_BaP_uM.(Intercept) | -0.93 |  |  |  |  |  |
|  |  |  | sd_BaP_uM | 0.01 |  |  |  |  |  |
|  |  | Residual | sd_Observation | 0.81 |  |  |  |  |  |
| Simpson | fixed |  | (Intercept) | 0.62 | 0.04 | 407 | 14.85 | 0 | * |
|  |  |  | BaP µM | 0 | 0 | 407 | 0.72 | 0.47 |  |
|  | ran_pars | SARL_plateID | sd_(Intercept) | 0.09 |  |  |  |  |  |
|  |  |  | cor_BaP_uM.(Intercept) | -0.91 |  |  |  |  |  |
|  |  |  | sd_BaP_uM | 0 |  |  |  |  |  |
|  |  | Residual | sd_Observation | 0.22 |  |  |  |  |  |
| Phylogenetic | fixed |  | (Intercept) | 6.52 | 0.24 | 407 | 26.71 | 0 | * |
|  |  |  | BaP µM | 0.09 | 0.04 | 407 | 2.2 | 0.028 | * |
|  | ran_pars | SARL_plateID | sd_(Intercept) | 0.01 |  |  |  |  |  |
|  |  |  | cor_BaP_uM.(Intercept) | -0.08 |  |  |  |  |  |
|  |  |  | sd_BaP_uM | 0 |  |  |  |  |  |
|  |  | Residual | sd_Observation | 3.43 |  |  |  |  |  |
| Observed | fixed |  | (Intercept) | 29.88 | 1.76 | 407 | 16.97 | 0 | * |
|  |  |  | BaP µM | 0.84 | 0.35 | 407 | 2.38 | 0.018 | * |
|  | ran_pars | SARL_plateID | sd_(Intercept) | 1.49 |  |  |  |  |  |
|  |  |  | cor_BaP_uM.(Intercept) | -0.89 |  |  |  |  |  |
|  |  |  | sd_BaP_uM | 0.49 |  |  |  |  |  |
|  |  | Residual | sd_Observation | 23.22 |  |  |  |  |  |

Supplemental Table 4: PERMANOVA models testing the association between BaP exposure and larval microbiome beta-diversity metrics.

| **Beta.metric** | **Term** | **Df** | **SumOfSqs** | **Statistic** | **P.value** |
| --- | --- | --- | --- | --- | --- |
| Canberra | BaP_uM | 1 | 0.5 | 1.1 | 0.18 |
|  | Residual | 411 | 189.26 |  |  |
| Sørensen | BaP_uM | 1 | 0.39 | 1.1 | 0.26 |
|  | Residual | 411 | 143.41 |  |  |
| Weighted UniFrac | BaP_uM | 1 | 0.02 | 1.06 | 0.36 |
|  | Residual | 411 | 8.55 |  |  |
| Unweighted UniFrac | BaP_uM | 1 | 0.44 | 1.35 | 0.09 |
|  | Residual | 411 | 135.78 |  |  |

Supplemental Table 5. A confusion matrix for the random forest classification model testing how well taxon abundances predicted BaP exposure. We ran this model for ASV-level data and “aggregated” data (i.e., higher-level taxonomic assignments (e.g., genus, family, class, etc.)).

| **ASV-only** | | | | | |
| --- | --- | --- | --- | --- | --- |
|  | Reference | | | | |
|  |  | uM0 | uM1 | uM5 | uM10 |
| Prediction | uM0 | 13 | 10 | 15 | 11 |
|  | uM1 | 5 | 6 | 6 | 7 |
|  | uM5 | 9 | 6 | 8 | 4 |
|  | uM10 | 3 | 9 | 3 | 11 |
|  |  |  |  | Accuracy (average) | 0.3016 |
|  |  |  |  | 95% CI | (0.2231, 0.3897) |
| **Aggregated** | | | | | |
|  | Reference | | | | |
|  |  | uM0 | uM1 | uM5 | uM10 |
| Prediction | uM0 | 8 | 6 | 11 | 9 |
|  | uM1 | 11 | 6 | 6 | 6 |
|  | uM5 | 4 | 8 | 8 | 5 |
|  | uM10 | 7 | 11 | 7 | 13 |
|  |  |  |  | Accuracy (average) | 0.2778 |
|  |  |  |  | 95% CI | (0.2017, 0.3646) |

Supplemental Table 6: Linear regression models testing the associations between LPR AUCs (larval activity) and BaP exposure, microbiome diversity.

| **Alpha.metric** | **Cycle** | **Effect** | **Group** | **Term** | **Estimate** | **Std.error** | **Df** | **Statistic** | **P.value** | **Sig** |
| --- | --- | --- | --- | --- | --- | --- | --- | --- | --- | --- |
| Chao1 | light | fixed |  | (Intercept) | 110.53 | 10.62 | 398 | 10.41 | 0 | * |
|  |  |  |  | BaP | 7.73 | 4.68 | 398 | 1.65 | 0.099 |  |
|  |  |  |  | BaP^2 | -0.91 | 0.45 | 398 | -2.02 | 0.044 | * |
|  |  | ran_pars | SARL_plateID | sd_(Intercept) | 17.59 |  |  |  |  |  |
|  |  |  | Residual | sd_Observation | 92.67 |  |  |  |  |  |
|  | dark | fixed |  | (Intercept) | 499.63 | 29.09 | 398 | 17.18 | 0 | * |
|  |  |  |  | BaP | 27.94 | 14.11 | 398 | 1.98 | 0.048 | * |
|  |  |  |  | BaP^2 | -2.93 | 1.35 | 398 | -2.17 | 0.031 | * |
|  |  | ran_pars | SARL_plateID | sd_(Intercept) | 41.82 |  |  |  |  |  |
|  |  |  | Residual | sd_Observation | 279.27 |  |  |  |  |  |
| Shannon | light | fixed |  | (Intercept) | 110.53 | 10.62 | 398 | 10.41 | 0 | * |
|  |  |  |  | BaP | 7.73 | 4.68 | 398 | 1.65 | 0.099 |  |
|  |  |  |  | BaP^2 | -0.91 | 0.45 | 398 | -2.02 | 0.044 | * |
|  |  | ran_pars | SARL_plateID | sd_(Intercept) | 17.59 |  |  |  |  |  |
|  |  |  | Residual | sd_Observation | 92.67 |  |  |  |  |  |
|  | dark | fixed |  | (Intercept) | 557.43 | 46.09 | 396 | 12.09 | 0 | * |
|  |  |  |  | BaP | 30.01 | 14.12 | 396 | 2.13 | 0.034 | * |
|  |  |  |  | BaP^2 | -4.52 | 1.55 | 396 | -2.92 | 0.004 | * |
|  |  |  |  | Shannon | -36.48 | 22.65 | 396 | -1.61 | 0.108 |  |
|  |  |  |  | BaP^2:Shannon | 0.81 | 0.39 | 396 | 2.08 | 0.038 | * |
|  |  | ran_pars | SARL_plateID | sd_(Intercept) | 40.51 |  |  |  |  |  |
|  |  |  | Residual | sd_Observation | 277.81 |  |  |  |  |  |
| Simpson | light | fixed |  | (Intercept) | 110.53 | 10.62 | 398 | 10.41 | 0 | * |
|  |  |  |  | BaP | 7.73 | 4.68 | 398 | 1.65 | 0.099 |  |
|  |  |  |  | BaP^2 | -0.91 | 0.45 | 398 | -2.02 | 0.044 | * |
|  |  | ran_pars | SARL_plateID | sd_(Intercept) | 17.59 |  |  |  |  |  |
|  |  |  | Residual | sd_Observation | 92.67 |  |  |  |  |  |
|  | dark | fixed |  | (Intercept) | 499.63 | 29.09 | 398 | 17.18 | 0 | * |
|  |  |  |  | BaP | 27.94 | 14.11 | 398 | 1.98 | 0.048 | * |
|  |  |  |  | BaP^2 | -2.93 | 1.35 | 398 | -2.17 | 0.031 | * |
|  |  | ran_pars | SARL_plateID | sd_(Intercept) | 41.82 |  |  |  |  |  |
|  |  |  | Residual | sd_Observation | 279.27 |  |  |  |  |  |
| Phylogenetic | light | fixed |  | (Intercept) | 110.53 | 10.62 | 398 | 10.41 | 0 | * |
|  |  |  |  | BaP | 7.73 | 4.68 | 398 | 1.65 | 0.099 |  |
|  |  |  |  | BaP^2 | -0.91 | 0.45 | 398 | -2.02 | 0.044 | * |
|  |  | ran_pars | SARL_plateID | sd_(Intercept) | 17.59 |  |  |  |  |  |
|  |  |  | Residual | sd_Observation | 92.67 |  |  |  |  |  |
|  | dark | fixed |  | (Intercept) | 499.63 | 29.09 | 398 | 17.18 | 0 | * |
|  |  |  |  | BaP | 27.94 | 14.11 | 398 | 1.98 | 0.048 | * |
|  |  |  |  | BaP^2 | -2.93 | 1.35 | 398 | -2.17 | 0.031 | * |
|  |  | ran_pars | SARL_plateID | sd_(Intercept) | 41.82 |  |  |  |  |  |
|  |  |  | Residual | sd_Observation | 279.27 |  |  |  |  |  |
| Observed | light | fixed |  | (Intercept) | 110.53 | 10.62 | 398 | 10.41 | 0 | * |
|  |  |  |  | BaP | 7.73 | 4.68 | 398 | 1.65 | 0.099 |  |
|  |  |  |  | BaP^2 | -0.91 | 0.45 | 398 | -2.02 | 0.044 | * |
|  |  | ran_pars | SARL_plateID | sd_(Intercept) | 17.59 |  |  |  |  |  |
|  |  |  | Residual | sd_Observation | 92.67 |  |  |  |  |  |
|  | dark | fixed |  | (Intercept) | 499.63 | 29.09 | 398 | 17.18 | 0 | * |
|  |  |  |  | BaP | 27.94 | 14.11 | 398 | 1.98 | 0.048 | * |
|  |  |  |  | BaP^2 | -2.93 | 1.35 | 398 | -2.17 | 0.031 | * |
|  |  | ran_pars | SARL_plateID | sd_(Intercept) | 41.82 |  |  |  |  |  |
|  |  |  | Residual | sd_Observation | 279.27 |  |  |  |  |  |

Supplemental Table 7: PERMANOVA models testing the association between beta-diversity scores and BaP exposure, LPR AUC scores.

| **Beta.metric** | **Cycle** | **Term** | **Df** | **SumOfSqs** | **Statistic** | **P.value** |
| --- | --- | --- | --- | --- | --- | --- |
| Canberra | light | LPR.light.auc | 1 | 0.56 | 1.228 | 0.11 |
|  |  | Residual | 403 | 185.47 |  |  |
|  | dark | LPR.dark.auc | 1 | 0.54 | 1.178 | 0.13 |
|  |  | Residual | 403 | 185.49 |  |  |
| Sørensen | light | LPR.light.auc | 1 | 0.61 | 1.753 | 0.07 |
|  |  | Residual | 403 | 140.31 |  |  |
|  | dark | LPR.dark.auc | 1 | 0.56 | 1.602 | 0.11 |
|  |  | Residual | 403 | 140.37 |  |  |
| Weighted UniFrac |  | BaP_uM | 1 | 0.02 | 1.129 | 0.38 |
|  |  | LPR.dark.auc |  | 0.03 | 1.351 | 0.3 |
|  |  | BaP_uM:LPR.dark.auc |  | 0.04 | 1.849 | 0.9 |
|  |  | Residual | 401 | 8.3 |  |  |
| Unweighted UniFrac | light | BaP_uM | 1 | 0.45 | 1.363 | 0.13 |
|  |  | LPR.light.auc |  | 0.4 | 1.225 | 0.21 |
|  |  | Residual | 402 | 132.85 |  |  |
|  | dark | BaP_uM | 1 | 0.45 | 1.364 | 0.11 |
|  |  | LPR.dark.auc |  | 0.43 | 1.311 | 0.14 |
|  |  | BaP_uM:LPR.dark.auc |  | 0.39 | 1.196 | 0.21 |
|  |  | Residual | 401 | 132.43 |  |  |

Supplemental Table 8. Random forest regression model metrics, using taxon abundances to predict either light or dark cycle AUCs. These were calculated using taxon abundances at the ASV level and "aggregated" (i.e., higher-level taxonomic assignments (e.g., genus, family, class, etc.)).

| **Taxa set** | **Cycle** | **RMSE (root mean squared error) [uM BaP]** |
| --- | --- | --- |
| ASV only | light | 86.52266 |
| ASV only | dark | 286.92681 |
| aggregated | light | 86.64777 |
| aggregated | dark | 286.52175 |

Supplemental Table 9: Linear mixed-effects regression models predicting LPR AUC values from individual taxon abundances and BaP exposure. Interactions terms between taxon abundance and BaP exposure were only included if that term significantly improved the model.

| **Taxon** | **Cycle** | **Effect** | **Group** | **Term** | **Estimate** | **Std.error** | **Df** | **Statistic** | **P.value** | **Sig** | **Mod.pval** |
| --- | --- | --- | --- | --- | --- | --- | --- | --- | --- | --- | --- |
| ASV00006 | light | fixed |  | (Intercept) | 102.32 | 7.95 | 399 | 12.87 | 0 | * | 0 |
|  |  |  |  | BaP | 6.71 | 4.65 | 399 | 1.44 | 0.15 |  |  |
|  |  |  |  | BaP^2 | -0.8 | 0.44 | 399 | -1.8 | 0.072 |  |  |
|  |  |  |  | ASV00006 | 0.02 | 0 | 399 | 4.29 | 0 | * |  |
|  |  | ran_pars | SARL_plateID | sd_(Intercept) | 1.64 |  |  |  |  |  |  |
|  |  |  |  | cor_BaP_uM.(Intercept) | -0.54 |  |  |  |  |  |  |
|  |  |  |  | sd_BaP | 1.22 |  |  |  |  |  |  |
|  |  |  | Residual | sd_Observation | 92.02 |  |  |  |  |  |  |
| ASV00071 |  | fixed |  | (Intercept) | 107.73 | 9.97 | 399 | 10.81 | 0 | * | 0.008 |
|  |  |  |  | BaP | 7.75 | 4.69 | 399 | 1.65 | 0.099 |  |  |
|  |  |  |  | BaP^2 | -0.9 | 0.45 | 399 | -2.01 | 0.045 | * |  |
|  |  |  |  | ASV00071 | 0.09 | 0.03 | 399 | 2.66 | 0.008 | * |  |
|  |  | ran_pars | SARL_plateID | sd_(Intercept) | 15.04 |  |  |  |  |  |  |
|  |  |  |  | cor_BaP_uM.(Intercept) | -0.26 |  |  |  |  |  |  |
|  |  |  |  | sd_BaP | 1.59 |  |  |  |  |  |  |
|  |  |  | Residual | sd_Observation | 92.05 |  |  |  |  |  |  |
| ASV00067 |  | fixed |  | (Intercept) | 108.61 | 9.51 | 398 | 11.42 | 0 | * | 0.003 |
|  |  |  |  | BaP | 5.81 | 4.68 | 398 | 1.24 | 0.215 |  |  |
|  |  |  |  | BaP^2 | -0.68 | 0.45 | 398 | -1.53 | 0.127 |  |  |
|  |  |  |  | ASV00067 | 0.31 | 0.1 | 398 | 2.92 | 0.004 | * |  |
|  |  |  |  | BaP^2:ASV00067 | 0 | 0 | 398 | -3 | 0.003 | * |  |
|  |  | ran_pars | SARL_plateID | sd_(Intercept) | 13.32 |  |  |  |  |  |  |
|  |  |  |  | cor_BaP_uM.(Intercept) | -0.15 |  |  |  |  |  |  |
|  |  |  |  | sd_BaP | 1.43 |  |  |  |  |  |  |
|  |  |  | Residual | sd_Observation | 91.93 |  |  |  |  |  |  |
| ASV00154 |  | fixed |  | (Intercept) | 110.18 | 10.54 | 399 | 10.45 | 0 | * | 0.007 |
|  |  |  |  | BaP | 5.65 | 4.67 | 399 | 1.21 | 0.227 |  |  |
|  |  |  |  | BaP^2 | -0.71 | 0.45 | 399 | -1.6 | 0.111 |  |  |
|  |  |  |  | ASV00154 | 0.44 | 0.16 | 399 | 2.69 | 0.007 | * |  |
|  |  | ran_pars | SARL_plateID | sd_(Intercept) | 17.41 |  |  |  |  |  |  |
|  |  |  |  | cor_BaP_uM.(Intercept) | -0.25 |  |  |  |  |  |  |
|  |  |  |  | sd_BaP | 1.26 |  |  |  |  |  |  |
|  |  |  | Residual | sd_Observation | 91.95 |  |  |  |  |  |  |
| p__Bdellovibrionota.c__Bdellovibrionia | dark | fixed |  | (Intercept) | 503.32 | 28.04 | 397 | 17.95 | 0 | * | 0.046 |
|  |  |  |  | BaP | 28.39 | 14.12 | 397 | 2.01 | 0.045 | * |  |
|  |  |  |  | BaP^2 | -2.93 | 1.35 | 397 | -2.17 | 0.03 | * |  |
|  |  |  |  | p__Bdellovibrionotac__Bdellovibrionia | -0.48 | 0.24 | 397 | -2 | 0.046 | * |  |
|  |  | ran_pars | SARL_plateID | sd_(Intercept) | 37.33 |  |  |  |  |  |  |
|  |  |  |  | cor_BaP_uM.(Intercept) | 0.06 |  |  |  |  |  |  |
|  |  |  |  | sd_BaP | 3.59 |  |  |  |  |  |  |
|  |  |  | Residual | sd_Observation | 277.57 |  |  |  |  |  |  |
| p__WPS-2 |  | fixed |  | (Intercept) | 503.53 | 27.17 | 397 | 18.53 | 0 | * | 0.04 |
|  |  |  |  | BaP | 28.06 | 14.12 | 397 | 1.99 | 0.048 | * |  |
|  |  |  |  | BaP^2 | -2.89 | 1.35 | 397 | -2.14 | 0.033 | * |  |
|  |  |  |  | p__WPS2 | -0.89 | 0.43 | 397 | -2.05 | 0.041 | * |  |
|  |  | ran_pars | SARL_plateID | sd_(Intercept) | 33.24 |  |  |  |  |  |  |
|  |  |  |  | cor_BaP_uM.(Intercept) | 0.19 |  |  |  |  |  |  |
|  |  |  |  | sd_BaP | 3.46 |  |  |  |  |  |  |
|  |  |  | Residual | sd_Observation | 277.68 |  |  |  |  |  |  |
| p__Proteobacteria.c__Gammaproteobacteria.o__Alteromonadales.f__Shewanellaceae |  | fixed |  | (Intercept) | 475.49 | 31.52 | 397 | 15.08 | 0 | * | 0.014 |
|  |  |  |  | BaP | 29.68 | 14.04 | 397 | 2.11 | 0.035 | * |  |
|  |  |  |  | BaP^2 | -2.98 | 1.34 | 397 | -2.22 | 0.027 | * |  |
|  |  |  |  | p__Proteobacteriac__Gammaproteobacteriao__Alteromonadalesf__Shewanellaceae | 0.02 | 0.01 | 397 | 2.5 | 0.013 | * |  |
|  |  | ran_pars | SARL_plateID | sd_(Intercept) | 45.98 |  |  |  |  |  |  |
|  |  |  |  | cor_BaP_uM.(Intercept) | -0.06 |  |  |  |  |  |  |
|  |  |  |  | sd_BaP | 1.86 |  |  |  |  |  |  |
|  |  |  | Residual | sd_Observation | 276.77 |  |  |  |  |  |  |
| ASV00003 |  | fixed |  | (Intercept) | 482.11 | 29.57 | 397 | 16.31 | 0 | * | 0.024 |
|  |  |  |  | BaP | 29.18 | 14.07 | 397 | 2.07 | 0.039 | * |  |
|  |  |  |  | BaP^2 | -2.99 | 1.35 | 397 | -2.22 | 0.027 | * |  |
|  |  |  |  | ASV00003 | 0.03 | 0.01 | 397 | 2.27 | 0.024 | * |  |
|  |  | ran_pars | SARL_plateID | sd_(Intercept) | 40.04 |  |  |  |  |  |  |
|  |  |  |  | cor_BaP_uM.(Intercept) | 0 |  |  |  |  |  |  |
|  |  |  |  | sd_BaP | 2.2 |  |  |  |  |  |  |
|  |  |  | Residual | sd_Observation | 277.38 |  |  |  |  |  |  |
| p__Proteobacteria.c__Alphaproteobacteria.o__Rhizobiales.f__Xanthobacteraceae.g__Afipia |  | fixed |  | (Intercept) | 492.11 | 26.51 | 397 | 18.56 | 0 | * | 0.035 |
|  |  |  |  | BaP | 27.15 | 14.15 | 397 | 1.92 | 0.056 |  |  |
|  |  |  |  | BaP^2 | -2.83 | 1.35 | 397 | -2.1 | 0.037 | * |  |
|  |  |  |  | p__Proteobacteriac__Alphaproteobacteriao__Rhizobialesf__Xanthobacteraceaeg__Afipia | 0.58 | 0.27 | 397 | 2.11 | 0.035 | * |  |
|  |  | ran_pars | SARL_plateID | sd_(Intercept) | 29.5 |  |  |  |  |  |  |
|  |  |  |  | cor_BaP_uM.(Intercept) | 0.36 |  |  |  |  |  |  |
|  |  |  |  | sd_BaP | 3.97 |  |  |  |  |  |  |
|  |  |  | Residual | sd_Observation | 277.56 |  |  |  |  |  |  |
| p__Proteobacteria.c__Alphaproteobacteria.o__Rhodospirillales |  | fixed |  | (Intercept) | 502.48 | 28.59 | 396 | 17.57 | 0 | * | 0.005 |
|  |  |  |  | BaP | 30.79 | 14.07 | 396 | 2.19 | 0.029 | * |  |
|  |  |  |  | BaP^2 | -3.27 | 1.34 | 396 | -2.44 | 0.015 | * |  |
|  |  |  |  | p__Proteobacteriac__Alphaproteobacteriao__Rhodospirillales | -0.16 | 0.05 | 396 | -3.13 | 0.002 | * |  |
|  |  |  |  | BaP^2:p__Proteobacteriac__Alphaproteobacteriao__Rhodospirillales | 0 | 0 | 396 | 2.85 | 0.005 | * |  |
|  |  | ran_pars | SARL_plateID | sd_(Intercept) | 40.53 |  |  |  |  |  |  |
|  |  |  |  | cor_BaP_uM.(Intercept) | 0.04 |  |  |  |  |  |  |
|  |  |  |  | sd_BaP | 4.23 |  |  |  |  |  |  |
|  |  |  | Residual | sd_Observation | 275.22 |  |  |  |  |  |  |
| p__Firmicutes.c__Clostridia.o__Lachnospirales.f__Lachnospiraceae.g__Lachnospiraceae_Genus |  | fixed |  | (Intercept) | 505.82 | 27.4 | 396 | 18.46 | 0 | * |  |
|  |  |  |  | BaP | 27.87 | 14.06 | 396 | 1.98 | 0.048 | * |  |
|  |  |  |  | BaP^2 | -3.15 | 1.35 | 396 | -2.34 | 0.02 | * |  |
|  |  |  |  | p__Firmicutesc__Clostridiao__Lachnospiralesf__Lachnospiraceaeg__Lachnospiraceae_Genus | -0.12 | 0.07 | 396 | -1.8 | 0.073 |  |  |
|  |  |  |  | BaP^2:p__Firmicutesc__Clostridiao__Lachnospiralesf__Lachnospiraceaeg__Lachnospiraceae_Genus | 0 | 0 | 396 | 2.82 | 0.005 | * |  |
|  |  | ran_pars | SARL_plateID | sd_(Intercept) | 33.65 |  |  |  |  |  |  |
|  |  |  |  | cor_BaP_uM.(Intercept) | 0.09 |  |  |  |  |  |  |
|  |  |  |  | sd_BaP | 2.82 |  |  |  |  |  |  |
|  |  |  | Residual | sd_Observation | 276.49 |  |  |  |  |  |  |
| p__Proteobacteria.c__Gammaproteobacteria.o__Burkholderiales.f__Comamonadaceae.g__Paucibacter |  | fixed |  | (Intercept) | 502.38 | 28.18 | 397 | 17.83 | 0 | * |  |
|  |  |  |  | BaP | 30.02 | 14.06 | 397 | 2.13 | 0.033 | * |  |
|  |  |  |  | BaP^2 | -3.09 | 1.34 | 397 | -2.3 | 0.022 | * |  |
|  |  |  |  | p__Proteobacteriac__Gammaproteobacteriao__Burkholderialesf__Comamonadaceaeg__Paucibacter | -1.75 | 0.62 | 397 | -2.81 | 0.005 | * |  |
|  |  | ran_pars | SARL_plateID | sd_(Intercept) | 38.67 |  |  |  |  |  |  |
|  |  |  |  | cor_BaP_uM.(Intercept) | 0.21 |  |  |  |  |  |  |
|  |  |  |  | sd_BaP | 3.61 |  |  |  |  |  |  |
|  |  |  | Residual | sd_Observation | 276.07 |  |  |  |  |  |  |
| p__Firmicutes.c__Clostridia |  | fixed |  | (Intercept) | 509.77 | 27.31 | 396 | 18.67 | 0 | * | 0.002 |
|  |  |  |  | BaP | 29.39 | 14.04 | 396 | 2.09 | 0.037 | * |  |
|  |  |  |  | BaP^2 | -3.36 | 1.35 | 396 | -2.49 | 0.013 | * |  |
|  |  |  |  | p__Firmicutesc__Clostridia | -0.06 | 0.02 | 396 | -2.47 | 0.014 | * |  |
|  |  |  |  | BaP^2:p__Firmicutesc__Clostridia | 0 | 0 | 396 | 3.12 | 0.002 | * |  |
|  |  | ran_pars | SARL_plateID | sd_(Intercept) | 33.05 |  |  |  |  |  |  |
|  |  |  |  | cor_BaP_uM.(Intercept) | 0.03 |  |  |  |  |  |  |
|  |  |  |  | sd_BaP | 2.87 |  |  |  |  |  |  |
|  |  |  | Residual | sd_Observation | 276 |  |  |  |  |  |  |
| p__Proteobacteria.c__Gammaproteobacteria.o__Burkholderiales.f__Oxalobacteraceae.g__Undibacterium |  | fixed |  | (Intercept) | 508.58 | 27.66 | 397 | 18.39 | 0 | * | 0.004 |
|  |  |  |  | BaP | 28.52 | 14.05 | 397 | 2.03 | 0.043 | * |  |
|  |  |  |  | BaP^2 | -2.98 | 1.34 | 397 | -2.22 | 0.027 | * |  |
|  |  |  |  | p__Proteobacteriac__Gammaproteobacteriao__Burkholderialesf__Oxalobacteraceaeg__Undibacterium | -0.58 | 0.2 | 397 | -2.9 | 0.004 | * |  |
|  |  | ran_pars | SARL_plateID | sd_(Intercept) | 35.6 |  |  |  |  |  |  |
|  |  |  |  | cor_BaP_uM.(Intercept) | 0.2 |  |  |  |  |  |  |
|  |  |  |  | sd_BaP | 3.73 |  |  |  |  |  |  |
|  |  |  | Residual | sd_Observation | 276.03 |  |  |  |  |  |  |
| p__Firmicutes.c__Clostridia.o__Lachnospirales.f__Lachnospiraceae.g__Blautia |  | fixed |  | (Intercept) | 505.85 | 27.81 | 396 | 18.19 | 0 | * | 0.003 |
|  |  |  |  | BaP | 28.4 | 14.01 | 396 | 2.03 | 0.043 | * |  |
|  |  |  |  | BaP^2 | -3.21 | 1.35 | 396 | -2.38 | 0.018 | * |  |
|  |  |  |  | p__Firmicutesc__Clostridiao__Lachnospiralesf__Lachnospiraceaeg__Blautia | -0.21 | 0.12 | 396 | -1.78 | 0.075 |  |  |
|  |  |  |  | BaP^2:p__Firmicutesc__Clostridiao__Lachnospiralesf__Lachnospiraceaeg__Blautia | 0.01 | 0 | 396 | 3.02 | 0.003 | * |  |
|  |  | ran_pars | SARL_plateID | sd_(Intercept) | 35.73 |  |  |  |  |  |  |
|  |  |  |  | cor_BaP_uM.(Intercept) | 0.01 |  |  |  |  |  |  |
|  |  |  |  | sd_BaP | 1.12 |  |  |  |  |  |  |
|  |  |  | Residual | sd_Observation | 276.45 |  |  |  |  |  |  |
| p__Fusobacteriota.c__Fusobacteriia |  | fixed |  | (Intercept) | 502.81 | 27.49 | 397 | 18.29 | 0 | * | 0.043 |
|  |  |  |  | BaP | 28.02 | 14.12 | 397 | 1.98 | 0.048 | * |  |
|  |  |  |  | BaP^2 | -2.91 | 1.35 | 397 | -2.16 | 0.031 | * |  |
|  |  |  |  | p__Fusobacteriotac__Fusobacteriia | -0.12 | 0.06 | 397 | -2.02 | 0.045 | * |  |
|  |  | ran_pars | SARL_plateID | sd_(Intercept) | 34.96 |  |  |  |  |  |  |
|  |  |  |  | cor_BaP_uM.(Intercept) | 0.26 |  |  |  |  |  |  |
|  |  |  |  | sd_BaP | 3.62 |  |  |  |  |  |  |
|  |  |  | Residual | sd_Observation | 277.53 |  |  |  |  |  |  |
| p__Firmicutes.c__Clostridia.o__Lachnospirales.f__Lachnospiraceae |  | fixed |  | (Intercept) | 509.04 | 27.43 | 396 | 18.56 | 0 | * | 0.002 |
|  |  |  |  | BaP | 28.88 | 14.03 | 396 | 2.06 | 0.04 | * |  |
|  |  |  |  | BaP^2 | -3.28 | 1.35 | 396 | -2.44 | 0.015 | * |  |
|  |  |  |  | p__Firmicutesc__Clostridiao__Lachnospiralesf__Lachnospiraceae | -0.07 | 0.03 | 396 | -2.29 | 0.023 | * |  |
|  |  |  |  | BaP^2:p__Firmicutesc__Clostridiao__Lachnospiralesf__Lachnospiraceae | 0 | 0 | 396 | 3.05 | 0.002 | * |  |
|  |  | ran_pars | SARL_plateID | sd_(Intercept) | 33.55 |  |  |  |  |  |  |
|  |  |  |  | cor_BaP_uM.(Intercept) | 0.01 |  |  |  |  |  |  |
|  |  |  |  | sd_BaP | 2.51 |  |  |  |  |  |  |
|  |  |  | Residual | sd_Observation | 276.26 |  |  |  |  |  |  |
| ASV02217 |  | fixed |  | (Intercept) | 502.78 | 28.05 | 396 | 17.92 | 0 | * | 0.039 |
|  |  |  |  | BaP | 27.56 | 14.12 | 396 | 1.95 | 0.052 |  |  |
|  |  |  |  | BaP^2 | -2.97 | 1.35 | 396 | -2.2 | 0.029 | * |  |
|  |  |  |  | ASV02217 | -54.54 | 33.31 | 396 | -1.64 | 0.102 |  |  |
|  |  |  |  | BaP^2:ASV02217 | 1.61 | 0.78 | 396 | 2.06 | 0.04 | * |  |
|  |  | ran_pars | SARL_plateID | sd_(Intercept) | 37.28 |  |  |  |  |  |  |
|  |  |  |  | cor_BaP_uM.(Intercept) | 0.17 |  |  |  |  |  |  |
|  |  |  |  | sd_BaP | 3.16 |  |  |  |  |  |  |
|  |  |  | Residual | sd_Observation | 277.28 |  |  |  |  |  |  |
| p__Actinobacteriota.c__Actinobacteria.o__Frankiales.f__Nakamurellaceae |  | fixed |  | (Intercept) | 495.59 | 29.08 | 396 | 17.04 | 0 | * | 0.033 |
|  |  |  |  | BaP | 27.09 | 14.06 | 396 | 1.93 | 0.055 |  |  |
|  |  |  |  | BaP^2 | -2.81 | 1.35 | 396 | -2.09 | 0.037 | * |  |
|  |  |  |  | p__Actinobacteriotac__Actinobacteriao__Frankialesf__Nakamurellaceae | 5.66 | 2.16 | 396 | 2.63 | 0.009 | * |  |
|  |  |  |  | BaP^2:p__Actinobacteriotac__Actinobacteriao__Frankialesf__Nakamurellaceae | -0.06 | 0.03 | 396 | -2.14 | 0.033 | * |  |
|  |  | ran_pars | SARL_plateID | sd_(Intercept) | 42.11 |  |  |  |  |  |  |
|  |  |  |  | cor_BaP_uM.(Intercept) | 0.02 |  |  |  |  |  |  |
|  |  |  |  | sd_BaP | 2.68 |  |  |  |  |  |  |
|  |  |  | Residual | sd_Observation | 276.49 |  |  |  |  |  |  |
| ASV00132 |  | fixed |  | (Intercept) | 500.28 | 27.99 | 396 | 17.88 | 0 | * | 0.037 |
|  |  |  |  | BaP | 28.83 | 14.21 | 396 | 2.03 | 0.043 | * |  |
|  |  |  |  | BaP^2 | -3.1 | 1.36 | 396 | -2.28 | 0.023 | * |  |
|  |  |  |  | ASV00132 | -0.63 | 0.55 | 396 | -1.15 | 0.25 |  |  |
|  |  |  |  | BaP^2:ASV00132 | 0.02 | 0.01 | 396 | 2.1 | 0.036 | * |  |
|  |  | ran_pars | SARL_plateID | sd_(Intercept) | 37.29 |  |  |  |  |  |  |
|  |  |  |  | cor_BaP_uM.(Intercept) | 0.02 |  |  |  |  |  |  |
|  |  |  |  | sd_BaP | 2.43 |  |  |  |  |  |  |
|  |  |  | Residual | sd_Observation | 277.56 |  |  |  |  |  |  |
| p__Proteobacteria.c__Alphaproteobacteria.o__Sphingomonadales.f__Sphingomonadaceae.g__Novosphingobium |  | fixed |  | (Intercept) | 499.69 | 27.2 | 397 | 18.37 | 0 | * | 0.019 |
|  |  |  |  | BaP | 30.08 | 14.12 | 397 | 2.13 | 0.034 | * |  |
|  |  |  |  | BaP^2 | -3.1 | 1.35 | 397 | -2.3 | 0.022 | * |  |
|  |  |  |  | p__Proteobacteriac__Alphaproteobacteriao__Sphingomonadalesf__Sphingomonadaceaeg__Novosphingobium | -0.83 | 0.36 | 397 | -2.33 | 0.02 | * |  |
|  |  | ran_pars | SARL_plateID | sd_(Intercept) | 34 |  |  |  |  |  |  |
|  |  |  |  | cor_BaP_uM.(Intercept) | 0.24 |  |  |  |  |  |  |
|  |  |  |  | sd_BaP | 3.62 |  |  |  |  |  |  |
|  |  |  | Residual | sd_Observation | 277.13 |  |  |  |  |  |  |

Supplemental Table 10: Linear regression models testing the association between BaP exposure, microbiome treatment and LPR AUC values (larval activity) by light and dark cycles.

| **Model** | **Cycle** | **Term** | **Estimate** | **Std.error** | **Statistic** | **P.value** | **Sig** |
| --- | --- | --- | --- | --- | --- | --- | --- |
| 1st order | light | (Intercept) | 120.37 | 14.92 | 8.07 | 0 | * |
|  |  | BaP µM | 4.98 | 3.22 | 1.55 | 0.122 |  |
|  |  | CVZ | 22.83 | 20.78 | 1.1 | 0.272 |  |
|  |  | GF | 168.51 | 20.77 | 8.11 | 0 | * |
|  |  | BaP µM:CVZ | -5.23 | 4.12 | -1.27 | 0.204 |  |
|  |  | BaP µM:GF | -7.25 | 4.13 | -1.76 | 0.079 |  |
| 2nd order |  | (Intercept) | 102.98 | 14.39 | 7.16 | 0 | * |
|  |  | BaP µM | 20.48 | 6.02 | 3.4 | 0.001 | * |
|  |  | BaP µM^2 | -1.45 | 0.67 | -2.16 | 0.031 | * |
|  |  | CVZ | 25.98 | 18.21 | 1.43 | 0.154 |  |
|  |  | GF | 167.01 | 18.13 | 9.21 | 0 | * |
|  |  | BaP µM^2:CVZ | -0.71 | 0.41 | -1.73 | 0.084 |  |
|  |  | BaP µM^2:GF | -0.85 | 0.41 | -2.07 | 0.039 | * |
| 1st order | dark | (Intercept) | 361.76 | 24.22 | 14.94 | 0 | * |
|  |  | BaP µM | 5.59 | 5.24 | 1.07 | 0.286 |  |
|  |  | CVZ | -103.86 | 33.79 | -3.07 | 0.002 | * |
|  |  | GF | 52.89 | 33.77 | 1.57 | 0.118 |  |
|  |  | BaP µM:CVZ | -1.17 | 6.7 | -0.18 | 0.861 |  |
|  |  | BaP µM:GF | -3.81 | 6.72 | -0.57 | 0.571 |  |
| 2nd order |  | (Intercept) | 337.13 | 23.43 | 14.39 | 0 | * |
|  |  | BaP µM | 33.82 | 9.83 | 3.44 | 0.001 | * |
|  |  | BaP µM^2 | -2.96 | 1.1 | -2.7 | 0.007 | * |
|  |  | CVZ | -100.69 | 29.7 | -3.39 | 0.001 | * |
|  |  | GF | 47.8 | 29.58 | 1.62 | 0.106 |  |
|  |  | BaP µM^2:CVZ | -0.06 | 0.67 | -0.09 | 0.932 |  |
|  |  | BaP µM^2:GF | -0.17 | 0.67 | -0.26 | 0.795 |  |
